# Phosphoryl-ene Step-Growth Polymerization: A Route to Polyphosphinates

**DOI:** 10.1021/polymscitech.6c00013

**Published:** 2026-04-30

**Authors:** Chaowei Yue, Wenhui Mu, Yifan Li

**Affiliations:** School of Physical Science and Technology, 528837 ShanghaiTech University, Shanghai 201210, China

## Abstract

Phosphorus-containing polymers have found wide application. Consequently, their synthesis is of great interest. Existing methods, however, primarily afford polyphosphonates and polyphosphates. Herein, we report a step-growth ″phosphoryl-ene″ polymerization strategy for incorporating phosphinate directly into the polymer backbone. Readily accessible AB-type phosphoryl monomers undergo radical step-growth polyaddition to yield the target polyphosphinates. Furthermore, AA-type bis-phosphoryl monomers can be used in radical step-growth polymerization with dienes or anionic step-growth polymerization with di-esters. This strategy provides an unprecedented and versatile approach to phosphorus-containing polymers, opening promising possibilities for polyphosphinate-based functional materials.

## Graphical abstract

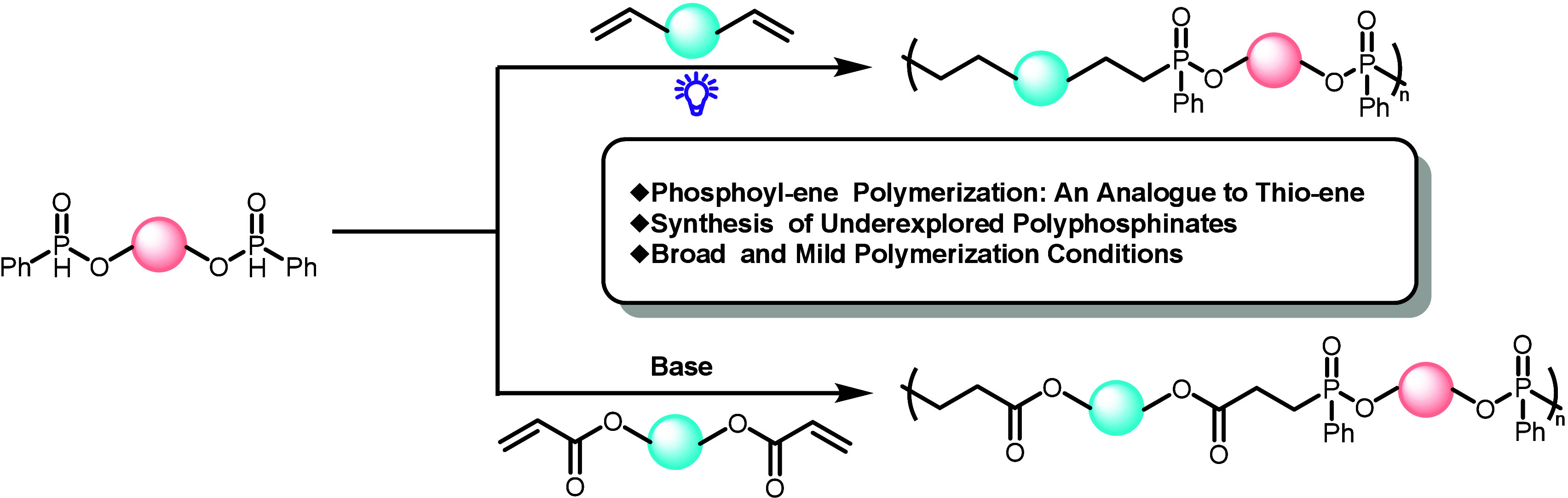

As a unique and functionally versatile subclass of heteroatom-containing polymers, materials with phosphate groups embedded directly within the main chain hold significant scientific and technological interest. The quintessential biological example of this architecture is found in natural genetic polymers, such as DNA and RNA, where the phosphate diester backbone is fundamental to both structural integrity and function. Beyond these biological blueprints, synthetic analogues have been engineered to exploit the distinctive chemical properties of phosphorus, leading to utility across a remarkably broad spectrum. Their applications span critical areas including advanced biomedical materials,^1^ effective metal chelation and sensing,^2^ high-performance flame-retardant coatings and additives,^3^ as well as robust corrosion inhibitors for metallic surfaces. This wide and impactful applicability, driven by the tunable reactivity, polarity, and thermal stability imparted by the phosphorus moiety, has consistently motivated intensive research into developing varied and efficient synthetic routes.

Based on the distinct functionalities of phosphorus, various monomers have been developed for use in polycondensation, polyaddition, and ring-opening polymerization.^4^ Synthetic strategies can be broadly categorized into two main directions. In the first approach, the phosphorus center is directly involved in the polymerization process (Figure 1a, red box). For example, Emrick demonstrated a polycondensation method using commercially available phenylphosphonic dichloride and diphenyl as monomers.^5^ To achieve polymers with greater control, cyclic phosphates or phosphonates are typically employed in ring-opening polymerization. Upon activation by a Brønsted base or Lewis base, polyphosphates or polyphosphonates with narrow molecular weight distributions can be obtained.^6–9^ The second approach relies on the transformation of functional groups attached to phosphorus-containing monomers, while the phosphorus center itself remains inert during polymerization. The most widely developed monomers in this category are phosphorus compounds bearing olefins, which can undergo polymerization via olefin metathesis, thiol–ene reactions, or Michael addition (Figure 1a, blue box).^10–17^ Although these methods have significantly advanced the synthesis of polyphosphates and polyphosphonates, polyphosphinates—a distinct subclass of phosphorus-containing polymers—remain relatively less studied.

**Figure 1:**
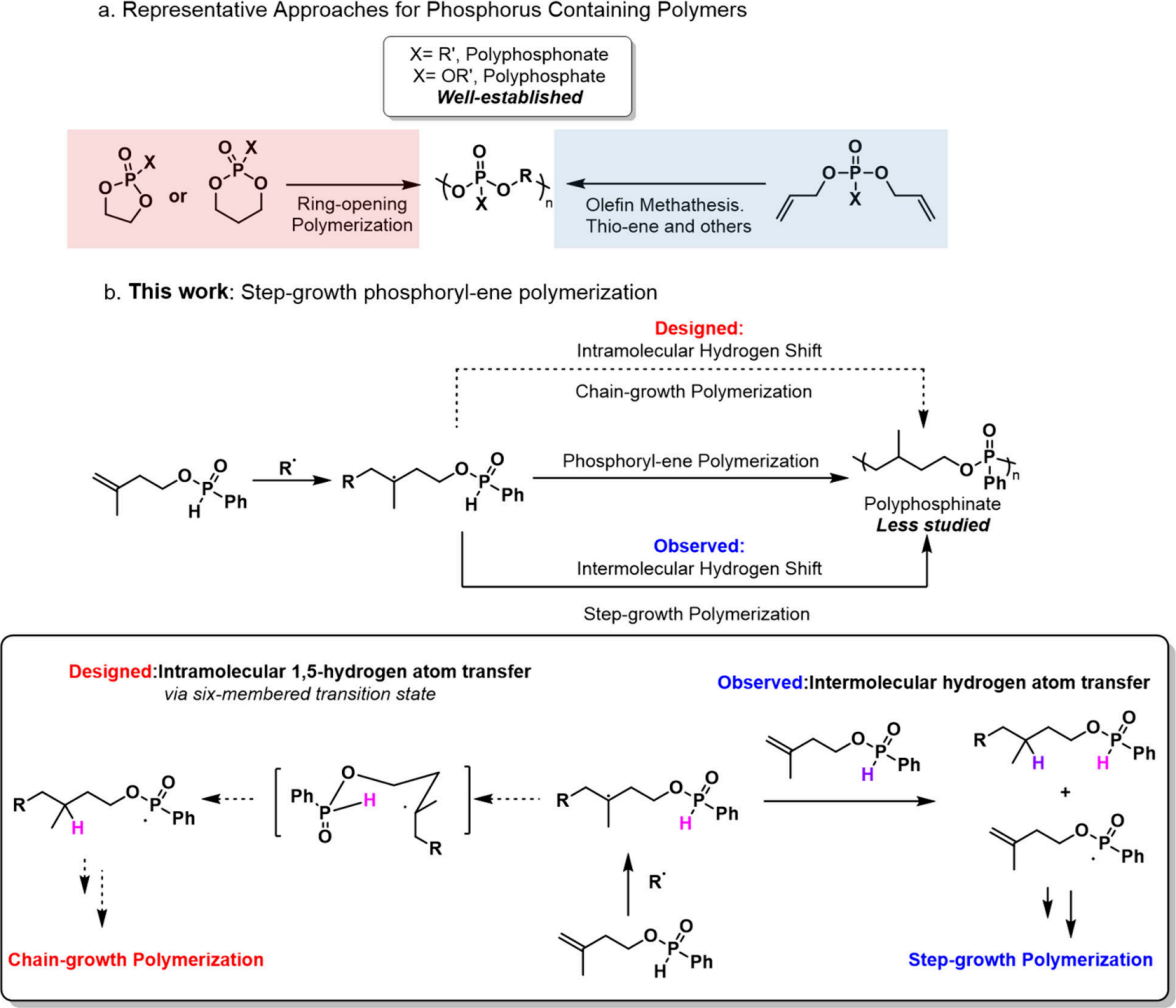
(a) Representative approaches for the synthesis of phosphorus-containing polymers. (b) This work: Phosphoryl-ene polymerization.

Our group specializes in designing 1-olefin and vinyl ether monomers, which are known to be incapable of radical homopolymerization, and in studying their behavior in both radical copolymerization and homopolymerization contexts.^18–24^ In 2024, we reported a radical homopolymerization of cyanated 1-olefins that proceeds via a 1,5- or 1,6-hydrogen atom transfer, enabling access to *C5* or *C6* polymers.^20^ In line with this research focus, we designed a phosphinated 1-olefin to investigate its radical homopolymerization. We hypothesized that, upon initiation, the nascent tertiary carbon radical would intramolecularly abstract a hydrogen atom from the phosphinate group. The resulting phosphorus-centered radical would then propagate through addition to the olefin functionality.^25^ Contrary to our hypothesis, kinetic studies revealed a step-growth polymerization mechanism. This result indicates that the critical hydrogen atom transfer occurs intermolecularly rather than intramolecularly. This observation inspired us to develop a phosphoryl-ene step-growth polymerization, conceptually analogous to the widely employed thio-ene polymerization. Herein, we report our recent efforts to synthesize phosphorus-containing polymers via radical phosphoryl-ene polymerization, utilizing either an AB-type phosphinated olefin monomer or AA-type diphosphinate monomers in combination with dienes. Furthermore, we have extended this methodology by employing AA-type diphosphinate monomers in a Michael-type addition polymerization with diesters. These approaches provide access to novel classes of polyphosphinates with unprecedented architectures. It is worth noting that, compared to well-established methods for synthesizing polyphosphonates and polyphosphates, the synthesis of polyphosphinates remains largely unexplored. This work thus opens new possibilities for the preparation of polyphosphinates, which may find potential applications in scale inhibition, corrosion control, dispersants, and other fields.

The phosphinate olefin monomer M1 was synthesized on a 4-gram scale in 78% yield from alcohol and dichlorophenylphosphine (see the Supporting Information for details). Polymerization was first investigated under thermal conditions using azobis­(isobutyronitrile) (AIBN) as the initiator in THF (2 M) at 60 °C. After 16 h, a conversion of 92% was achieved. The viscous reaction mixture was diluted with a minimal volume of dichloromethane and precipitated into diethyl ether, yielding a transparent gel. The resulting polymer exhibited good solubility in polar solvents such as dichloromethane, methanol, N,N-dimethylformamide (DMF), and dimethyl sulfoxide (DMSO). Size exclusion chromatography (SEC) analysis afforded a weight-average molecular weight (*M*_w, app_) of 4.0 k with a dispersity (*Đ*) of 1.33 (Table 1, entry 1). Conducting the polymerization at 80 °C led to quantitative conversion (Table 1, entry 2). We subsequently explored photo-initiated polymerization. Using 5 mol % 2,2-dimethoxy-2-phenylacetophenone (DMPA) as the initiator under 365 nm irradiation for 16 h gave 96% conversion (*p*= 0.90)­(Table 1, entry 3). Both DMF and dioxane were identified as suitable solvents, with DMPA proving to be an optimal photo-initiator. An attempt to increase the molecular weight by employing a higher monomer concentration (4 M in dioxane) resulted in the formation of an insoluble polymeric gel within 12 h (Table 1, entry 7). When the reaction was performed in DMF at the same concentration, conversions of 90% and 97% were obtained after 12 and 16 h, respectively. The product was partially soluble; SEC analysis of the soluble fraction yielded an *M*_w, app_ of 141.9 k (*p*= 0.99) (Table 1, entries 8 and 9). Further increasing the concentration to 8 M produced an insoluble polymer after 12 h of irradiation (Table 1, entry 10). Notably, oligomer formation was also observed under UV irradiation in the absence of a photo-initiator (Table 1, entry 11).

**Table 1:**
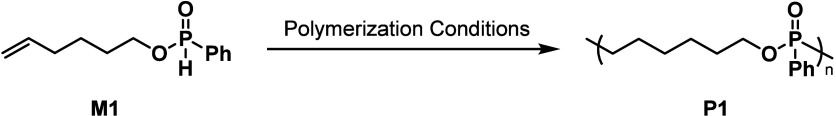
Screening of Polymerization Conditions

Entry	Initiator	Solvent	Cond.	Conc.	Time	Conv.	*p*	*M*_w,app_ ^a^	*Đ* ^a^
1	2 mol % AIBN	THF	60 °C	2M	16h	92%	0.93	4.0 k	1.33
2	2 mol % AIBN	THF	80 °C	2M	16h	99%	0.94	5.6 k	1.50
3	5 mol % DMPA	THF	365 nm	2M	12h	96%	0.90	3.3 k	1.48
4	5 mol % DMPA	Dioxane	365 nm	2M	12h	99%	0.96	18.7 k	3.23
5	5 mol % DMPA	DMF	365 nm	2M	16h	97%	0.96	13.9 k	2.48
6	5 mol % TPO	DMF	365 nm	2M	16h	85%	0.93	4.6 k	1.43
7	5 mol % DMPA	Dioxane	365 nm	4M	12h				*Insoluble*
8	5 mol % DMPA	DMF	365 nm	4M	16h	90%	0.97	22.4 k	2.99
9	5 mol % DMPA	DMF	365 nm	4M	19h	97%	0.99	141.6 k^b^	8.48^b^
10	5 mol % DMPA	DMF	365 nm	8M	12h				*Insoluble*
11	-	-	365 nm	-	16h	68%	0.93	4.3 k	1.27

a Apparent weight-average molar masses (*M* *_w,app_*) and dispersity (*Đ*) were determined by DMF SEC analysis calibrated to PMMA standards.

b The soluble part was analyzed. Cond.: condition; Conc.: concentration; Conv.: conversion; *p*: degree of reaction. TPO: 2,4,6-Trimethylbenzoyldiphenylphosphine oxide

Monomer M1 and polymer P1 were characterized by ^1^H NMR spectroscopy (Figure 2a). In the spectrum of M1, the phosphinate P–H protons resonate at 8.26 and 6.87 ppm. These signals are absent in the spectrum of P1, consistent with the loss of the P–H bond due to polymerization. The signal at 2.12–2.01 ppm in M1, assigned to the allylic protons, disappears in P1. Instead, a new signal emerges at 2.01–1.75 ppm, which is attributed to the phosphinate −CH_2_– protons. Kinetic studies revealed a progressive increase in molecular weight during the later stages of the reaction, indicative of a step-growth polymerization mechanism (Figure 2b).

**Figure 2:**
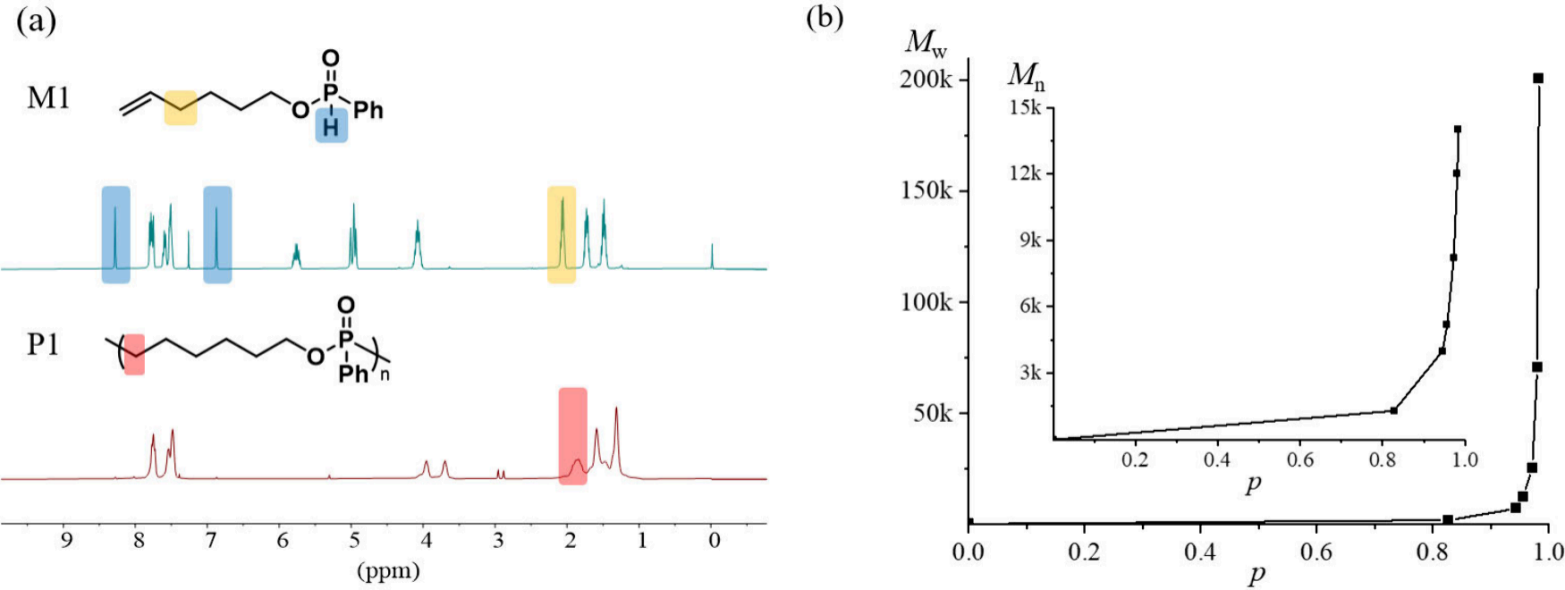
(a) ^1^H NMR spectral overlay of M1 and P1. (b) Kinetic profile of the polymerization reaction.

With the optimal conditions established, we subsequently investigated the scope of AB-type monomers. Several phosphoryl olefin monomers (M2–M4) were synthesized from the corresponding allylic alcohols and dichlorophenylphosphine. Polymerization of M2–M4 proceeded quantitatively, affording polyphosphinates with tunable backbone lengths (Figure 3, P2–P4). We then extended this approach to the synthesis of poly­(phosphine oxide). The monophenyl-substituted poly­(phosphine oxide) displayed limited solubility in THF and DMF but good solubility in methanol and chloroform, which precluded determination of its molecular weight by SEC analysis. However, introducing an additional phenyl ring into the polymer backbone improved solubility, allowing the preparation of diphenyl phosphine oxide-based polymers P6 and P7 in 82% and 88% yields, respectively. Furthermore, a diphosphinate monomer was designed for AA+BB-type polymerization. Copolymerization of M8a with hexa-1,5-diene (M8b) afforded P8 in 90% yield with an *M*_w,app_ of 33.1 k. The spacer length between phosphinate units could be tailored by copolymerization with decadiene, yielding P9 with an *M*_w,app_ of 331.3 k. Differential scanning calorimetry (DSC) and thermogravimetric analysis (TGA) were used to evaluate the thermal properties of these polyphosphinate. Depending on the main-chain structure, the polymers exhibit a wide range of glass transition temperatures (*T*_g_), from -17.6 °C to 61.4 °C. The low *T*_g_ values are attributed to the presence of flexible alkyl chains, while the high *T*_g_ observed for P6 arises from the phenyl moieties incorporated into the polymer backbone. Most of the polymers also display good thermal stability, with 5% weight loss temperatures (*T*_d_,_5%_) exceeding 160 °C. (Figures 3, S5–S8).

**Figure 3:**
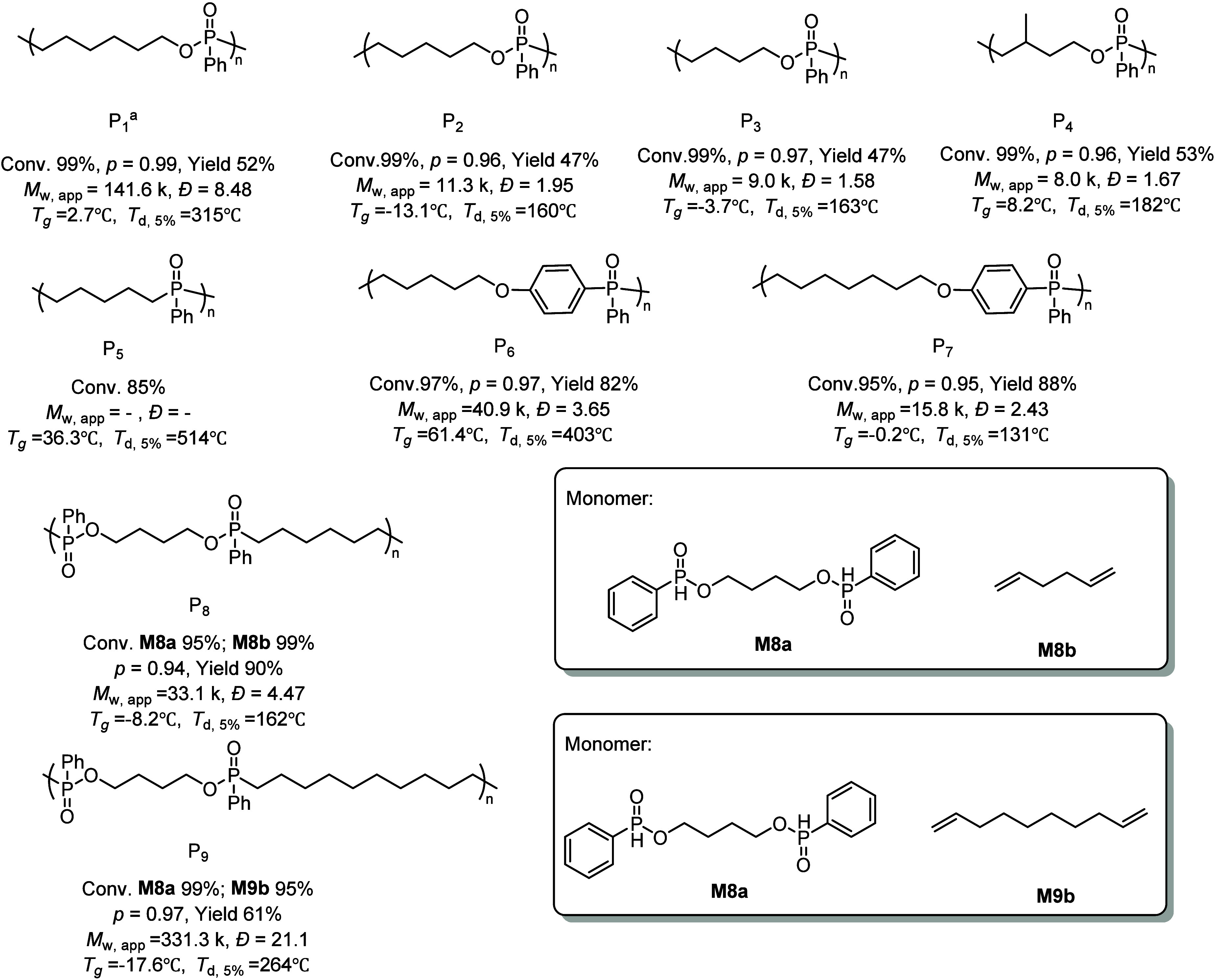
Scope of AB type phosphoryl-ene polymerization. The soluable part was analyzed.

Encouraged by this result, we subsequently examined the polymerization behavior of the diphosphinate with various diesters under anionic or radical conditions. We first attempted to copolymerize the diphosphinate with diester M10b under photo-irradiation. This reaction produced an insoluble gel, likely because M10b readily undergoes homopolymerization, forming a cross-linked polymer network. When the polymerization was conducted under anionic conditions using 1,8-Diazabicyclo[5.4.0]­undec-7-ene (DBU) as a base, polymer P10 was obtained with an *M*_w,app_ of 23.7 k. We then introduced a phenyl-substituted ester to inhibit this self-gelation. Under irradiation conditions, no polymer was formed. However, under anionic conditions, polymer P11 was obtained with 93% yield (Figure 4).

**Figure 4:**
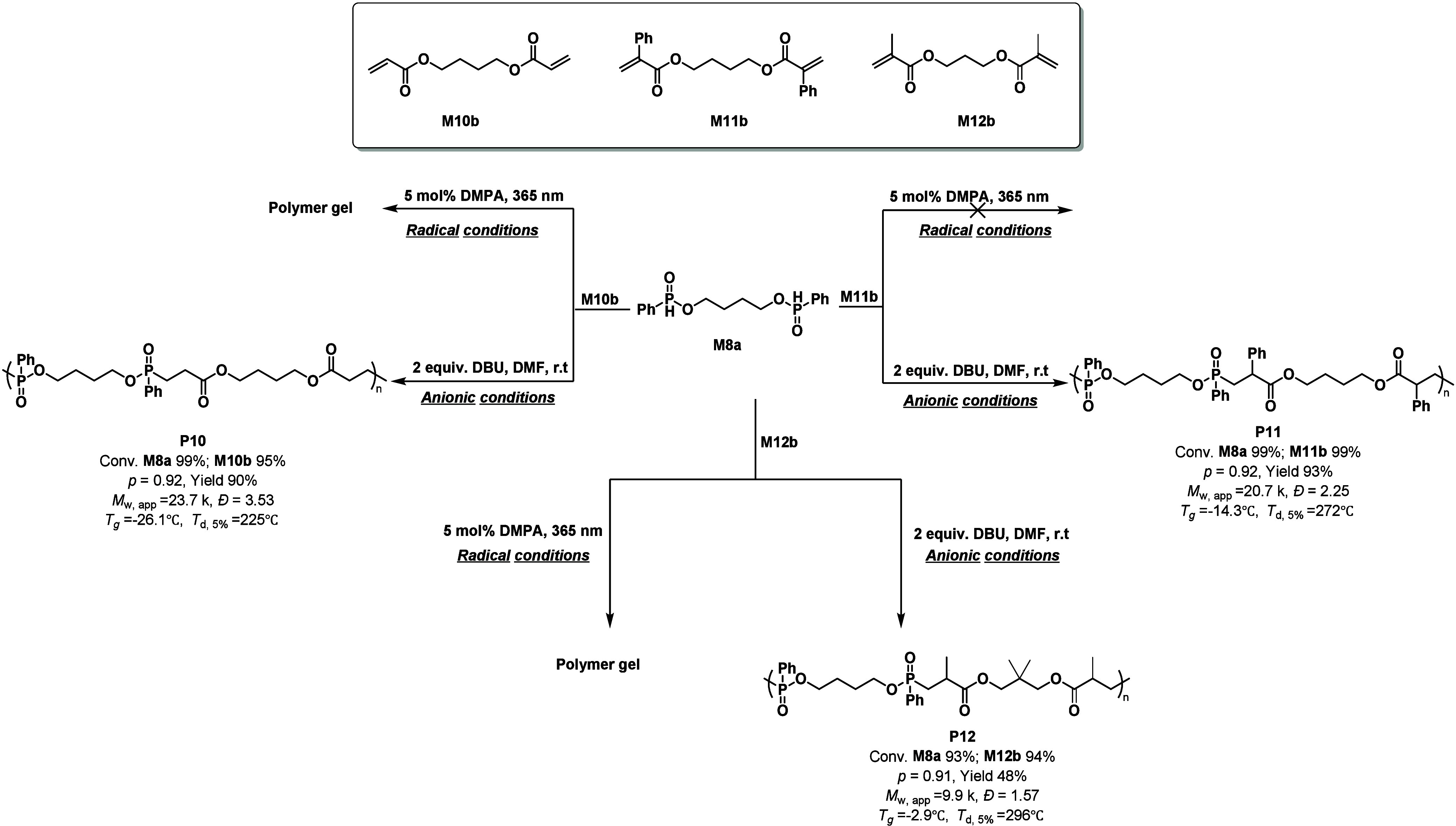
An-inic polymerization of diphophinates.

In summary, we have successfully developed a phosphoryl-ene polymerization as an analogue to thio-ene polymerization. The phosphinate monomer is readily synthesized from simple building blocks on a large scale. Its copolymerization with commercially available dienes or diesters proceeds efficiently under either photo-radical or anionic conditions. The resulting polyphosphinates, which are challenging to access via conventional routes, demonstrate the utility of this method. Given the broad applications of phosphorus-containing polymers, we believe this methodology will enable new possibilities for designing functional materials. Current work in our laboratory is focused on the design of new phosphorus-containing monomers and the study of their polymerization behavior.

## Supplementary Material



## Notes

Details for synthesis process, structural characterization, and experimental results (PDF)
