# Supplementary material for: Phosphoryl-ene Step-Growth Polymerization: A Route to Polyphosphinates
Source: Polym Sci Technol. 2026 Apr 30;2(7):482–7. doi: 10.1021/polymscitech.6c00013 (PMC13420099; doi:10.1021/polymscitech.6c00013)
Supplement: Supplementary file 1 [file ps6c00013_si_001.pdf]

Supporting Information

Phosphoryl-ene Step-Growth Polymerization: A Route to Polyphosphinates

Chaowei Yue, Wenhui Mu, Yifan Li*

School of Physical Science and Technology, ShanghaiTech University, 393 Middle Huaxia Road, Pudong, Shanghai 201210, China
*liyf3@shanghaitech.edu.cn;

General Information

Unless stated otherwise, all the chemical compounds and solvents were purchased from commercial suppliers at high quality and used without further purification. Reactions monitored by thin-layer chromatography (TLC) were performed on Energy Chemical silica gel GF254 TLC plates. UV light or $KMnO_4$ were used as visualization agents. Column chromatography for all small molecule purification was carried out on Leyan Silica Gel 200 X 300 mesh.

^{1}H-^{13}C spectra were conducted using Bruker Avance 400 MHz or 600 MHz instruments. Unless stated otherwise, spectrometers using residual chloroform (δ_H = 7.26 ppm and δ_C = 77.16 ppm), chemical shifts are reported as parts per million (ppm) referenced to residual protium or carbon of the solvents. Multiplicities were explained by the following abbreviations: s = singlet, d = doublet, t = triplet, q = quartet, p = pentet, m = multiplet, br = broad.

Weight-average molecular weights (M_w) and dispersity ($Đ$) of all polymers were determined by size exclusion chromatography (SEC). DMF SEC was performed using an SEC system equipped with Agilent 1260 Infinity II autosampler & quaternary pump, Shodex KD-803 + KD 805 tandem columns, and a Wyatt Optilab RI detector. HPLC-grade DMF with 10 mM LiBr was used as an eluent at 1.0 mL min^{-1} at 60 °C. Polymethyl methacrylate standards were used for relative molecular weight calibration for both systems. Samples were configured at 0.5-5 mg / mL and filtered through a 0.2 μm PTFE filter before injection. Data were analyzed by Agilent, Wyatt Astra Software, and OriginLab.

High-resolution mass spectra (HRMS) for new compounds were obtained from mass spectral facilities at ShanghaiTech University by Agilent Technologies 6230 TOF LC/MS spectrometer in electrospray ionization (ESI+ or ESI-) mode. Samples were configured at 1-10 μg/ ml (HPLC MeOH) and filtered through a 0.2 μm filter before injection.

UV LED (24 W, λ = 365 – 375 nm) was purchased from Wuhan Geao Chemical Technology Co., Ltd. was used for UV irradiation. A fan attached to the apparatus was used to maintain the reaction temperature at room temperature.

Differential scanning calorimetry (DSC) analyses were carried out (TA RCS90 DSC2500) at a heating rate of 10 ˚C / min. The onset of the T_g peak at the second heating cycle was adopted. Thermogravimetric analysis (TGA) of polymers was performed on a METTLER TOLEDO TGA3 Instruments at a heating rate of 10 ˚C/min under a 20 mL/min nitrogen flow. An isotherm process was employed at a temperature above the boiling point of the corresponding residual solvents of each sample. Traces were normalized to the remaining mass after the isotherm.

Monomer Synthesis

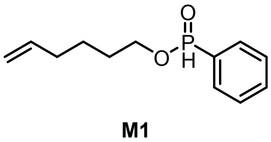

M1

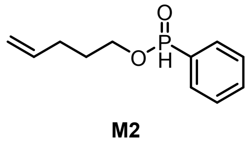

M2

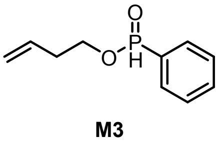

M3

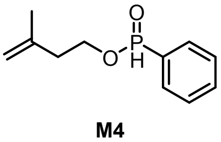

M4

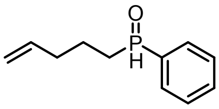

M5

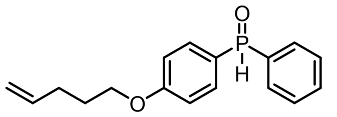

M6

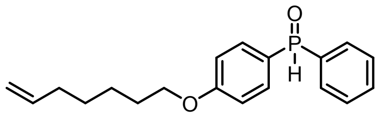

M7

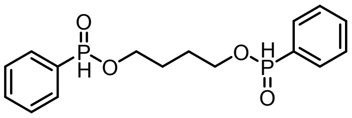

M8a

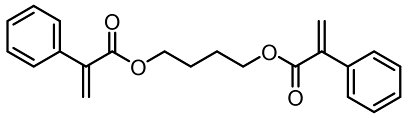

M11b

General procedure for the synthesis of **M1-M4** (**GP-I**):

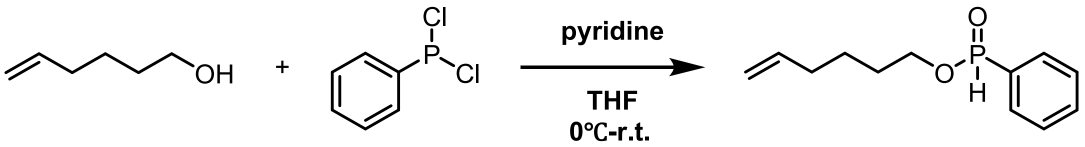

1 **2** **M1**

Procedure adapted from the literature.[1] To a 100 mL round-bottomed flask equipped with a stirring bar were added **2** (3.58 g, 20.0 mmol, 1.0 equiv.) and THF (20.0 mL) at 0˚C under N_2, followed by the slow addition of a mixture of **1** (2.0 g, 20.0 mmol, 1.0 equiv.) and pyridine (1.61 mL, 20.0 mmol, 1.0 equiv.) in THF (5.0 mL). The resulting mixture was stirred at room temperature overnight. The reaction was quenched with water, and the resulting mixture was extracted with ethyl acetate (30 mL × 3). The combined organic extracts were washed with brine (50 mL), dried over anhydrous Na_2SO_4, and concentrated under reduced pressure. The residue was purified by flash chromatography on silica gel (PE /EA=2:1-1:1) to afford **M1** as a colorless oil (3.5 g, 78% yield). **M1** was stored in a refrigerator under a nitrogen atmosphere. ^1H NMR (400 MHz, $CDCl_3$) δ 7.78 (ddt, *J* = 13.8, 8.3, 1.3 Hz, 2H), 7.64 – 7.45 (m, 3H), 7.51 (d, *J* = 564.1 Hz, 1H), 5.76 (ddtd, *J* = 17.0, 10.2, 6.6, 1.1 Hz, 1H), 5.04 – 4.90 (m, 2H), 4.16 – 3.99 (m, 2H), 2.06 (qd, *J* = 7.4, 2.7 Hz, 2H), 1.79 – 1.66 (m, 2H), 1.49 (p, *J* = 7.6 Hz,

2H). ^{13}C NMR (101 MHz, $CDCl_3$) δ 138.3, 133.2 (d, *J* = 2.9 Hz), 131.0 (d, *J* = 11.7 Hz), 130.0 (d, *J* = 132.0 Hz), 128.9 (d, *J* = 13.7 Hz), 115.1, 65.9 (d, *J* = 6.6 Hz), 33.2, 29.9 (d, *J* = 6.6 Hz), 24.9. ^{31}P NMR (162 MHz, $CDCl_3$) δ 25.0 (d, *J* = 563.3 Hz). HRMS (ESI) m/z calculated for $C_{12}H_{18}O_2P^+$ $[M+H]^+$ 225.1039 found 225.1040.

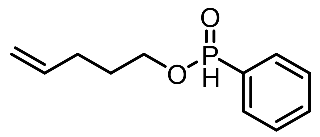

M2

According to **GP- Ⅰ**, **M2** was obtained as a colorless oil with a 80% yield. **M2** was stored in a refrigerator under a nitrogen atmosphere. ^{1}H NMR (400 MHz, $CDCl_3$) δ 7.83 – 7.73 (m, 2H), 7.58 (d, *J* = 563.3 Hz, 1H), 7.65 – 7.46 (m, 3H), 5.77 (ddt, *J* = 16.9, 10.2, 6.6 Hz, 1H), 5.08 – 4.94 (m, 2H), 4.17 – 4.00 (m, 2H), 2.23 – 2.11 (m, 2H), 1.83 (dt, *J* = 7.9, 6.6 Hz, 2H). ^{13}C NMR (101 MHz, $CDCl_3$) δ 137.2, 133.2 (d, *J* = 3.1 Hz), 131.0 (d, *J* = 11.7 Hz), 130.0 (d, *J* = 131.7 Hz), 128.9 (d, *J* = 13.7 Hz), 115.7, 65.3 (d, *J* = 6.4 Hz), 29.7, 29.6. ^{31}P NMR (162 MHz, $CDCl_3$) δ 25.1 (d, *J* = 563.8 Hz). HRMS (ESI) m/z calculated for $C_{11}H_{16}O_2P^+$ $[M+H]^+$ 211.0883 found 211.0883.

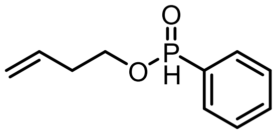

M3

According to **GP- Ⅰ**, **M3** was obtained as a colorless oil with a 75% yield. ^{1}H NMR (400 MHz, $CDCl_3$) δ 7.89 – 7.71 (m, 2H), 7.59 (d, *J* = 564.7 Hz, 1H), 7.56 (dtd, *J* = 37.9, 7.6, 2.6 Hz, 3H), 5.79 (ddt, *J* = 17.1, 10.4, 6.7 Hz, 1H), 5.19 – 5.07 (m, 2H), 4.13 (tdt, *J* = 14.8, 10.0, 6.9 Hz, 2H), 2.48 (q, *J* = 6.7 Hz, 2H). ^{13}C NMR (101 MHz, $CDCl_3$) δ 133.4, 133.3, 131.1 (d, *J* = 11.8 Hz), 129.9 (d, *J* = 132.1 Hz), 128.9 (d, *J* = 13.8 Hz), 118.1, 65.0 (d, *J* = 6.6 Hz), 35.0 (d, *J* = 6.6 Hz). ^{31}P NMR (162 MHz, $CDCl_3$) δ 25.1 (d, *J* = 562.1 Hz). HRMS (ESI) m/z calculated for $C_{10}H_{14}O_2P^+$ $[M+H]^+$ 197.0726 found 197.0726.

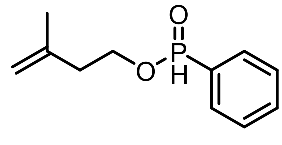

M4

According to **GP- Ⅰ**, **M4** was obtained as a colorless oil with a 84% yield. ^{1}H NMR (400 MHz, $CDCl_3$) δ 7.78 (ddt, *J* = 13.8, 6.8, 1.4 Hz, 2H), 7.60 (d, *J* = 564.4 Hz, 1H), 7.68 – 7.45 (m, 3H), 4.79 (m, 2H), 4.19 (dddt, *J* = 27.8, 10.1, 8.2, 6.8 Hz, 2H), 2.48 – 2.40 (m, 2H), 1.74 (s, 3H). ^{13}C NMR (101 MHz, $CDCl_3$) δ 141.1, 133.3, 131.1 (d, *J* = 11.9 Hz), 129.9 (d, *J* = 131.8 Hz), 128.9 (d, *J* = 13.7 Hz), 113.0, 64.0 (d, *J* = 6.6 Hz), 38.6 (d, *J* = 6.5 Hz), 22.6. ^{31}P NMR (162 MHz, $CDCl_3$) δ 25.1 (d, *J* = 564.8 Hz). HRMS (ESI) m/z calculated for $C_{11}H_{16}O_2P^+$ $[M+H]^+$ 211.0883 found 211.0882.

The synthesis of **M5**:

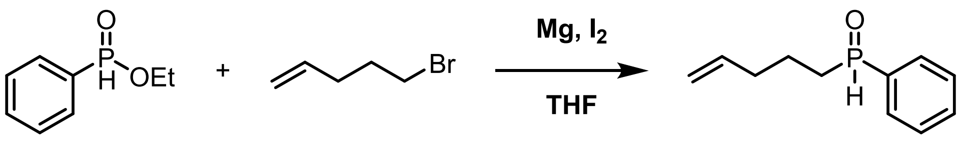

3 **4** **M5**

Procedure adapted from the literature.[2] To a 50 mL round bottom flask, **4** (0.98g, 6.6 mmol) was added along with Mg turnings (182 mg, 7.5 mmol), I_2 (catalytic) and THF (5 mL). The reaction was heated to reflux for 1 hour, and then the reaction was cooled to 0 °C and **3** (0.51 g, 3 mmol) was added dropwise with THF (2 mL). The reaction was heated to room temperature, mixed for 2 hours. After completion, the reaction was quenched with saturated aqueous NH_4Cl solution. The mixture was extracted with ethyl acetate (50 mL × 3), and the combined organic extracts were washed with brine (50 mL), dried over anhydrous Na_2SO_4, and concentrated under reduced pressure. The residue was purified by flash chromatography on silica gel (PE /EA=1:1) to afford **M5** as a colorless oil. (0.25 g, 43% yield). ^{1}H NMR (600 MHz, $CDCl_3$) δ 7.71 (ddt, J = 13.2, 6.8, 1.4 Hz, 2H), 7.60 – 7.49 (m, 3H), 7.51 (d, J = 463.9 Hz, 1H), 5.73 (ddt, J = 17.1, 10.3, 6.7 Hz, 1H), 5.05 – 4.94 (m, 2H), 2.17 (qt, J = 7.1, 1.4 Hz, 2H), 2.07 – 1.93 (m, 3H), 1.73 (m, 2H). ^{31}P NMR (243 MHz, $CDCl_3$) δ 27.61 (d, J = 463.5 Hz). The characterization data are consistent with the reported data.[3]

General procedure for the synthesis of **M6-M7** (**GP-Ⅱ**):

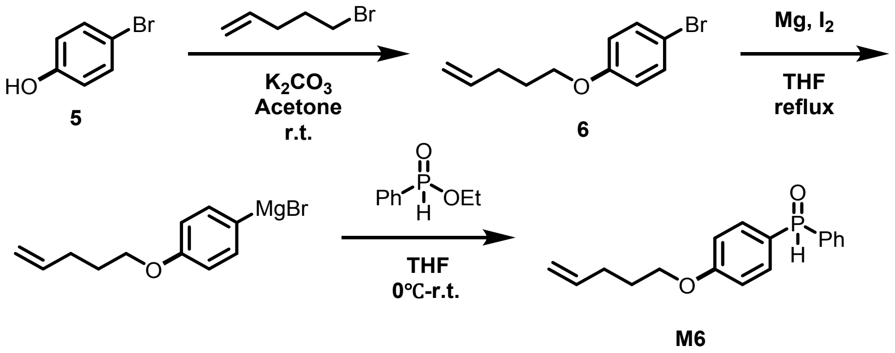

Procedure adapted from the literature.[1, 4] To a stirred solution of **5** (3.46g, 20mmol) in acetone (20 mL) under N_2 atmosphere was added K_2CO_3 (5.53 g, 40 mmol) and 5-bromopent-1-ene (3.28, 22 mmol). The reaction mixture was stirred at 60 °C for 12 h and filtered through Celite. The solvent was concentrated under reduced pressure, and the residue was purified by flash column chromatography on silica gel (PE /EA=30:1) to provide title compound **6** (3.94 g, 16.3 mmol, 82% yield) as a colorless oil. ^{1}H NMR (400 MHz, $CDCl_3$) δ 7.40 – 7.32 (m, 2H), 6.82 – 6.73 (m, 2H), 5.84 (ddt, J = 16.9, 10.2, 6.6 Hz, 1H), 5.11 – 4.96 (m, 2H), 3.93 (t, J = 6.4 Hz, 2H), 2.23 (tdd, J = 7.8, 6.1, 1.5

Hz, 2H), 1.88 (dt, *J* = 8.2, 6.6 Hz, 2H). The characterization data are consistent with the reported data.[4]

To a 50 mL round-bottomed flask equipped with a stirring bar under N_2 were added magnesium turnings (214 mg, 8.8 mmol) and a catalytic amount of I_2 in THF (2.0 mL). A solution of compound **6** (1.92 g, 8 mmol) in THF (20 mL) was then added dropwise at 68 °C. The resulting mixture was stirred at 68 °C for 1 hour and then cooled to 0 °C. The **3** (1.02 g, 6 mmol) was added, and the reaction mixture was stirred at room temperature for 2 hours. After completion, the reaction was quenched with saturated aqueous NH_4Cl solution. The mixture was extracted with ethyl acetate (50 mL × 3), and the combined organic extracts were washed with brine (50 mL), dried over anhydrous Na_2SO_4, and concentrated under reduced pressure. The residue was purified by flash chromatography on silica gel (PE /EA=2:1-1:1) to afford **M6** as a colorless oil (0.75 g, 44% yield). ^1H NMR (400 MHz, $CDCl_3$) δ 8.04 (d, *J* = 478.6 Hz, 1H), 7.75 – 7.45 (m, 7H), 7.02 – 6.96 (m, 2H), 5.83 (ddt, *J* = 16.9, 10.2, 6.6 Hz, 1H), 5.10 – 4.95 (m, 2H), 4.00 (t, *J* = 6.4 Hz, 2H), 2.28 – 2.17 (m, 2H), 1.89 (dt, *J* = 8.1, 6.5 Hz, 2H). ^{13}C NMR (101 MHz, $CDCl_3$) δ 162.6, 137.6, 132.7 (d, *J* = 13.0 Hz), 132.5, 131.5, 130.8 (d, *J* = 11.5 Hz), 128.9 (d, *J* = 12.8 Hz), 122.3 (d, *J* = 107.7 Hz), 115.5, 115.1 (d, *J* = 13.9 Hz), 67.4, 30.1, 28.3. ^{31}P NMR (162 MHz, $CDCl_3$) δ 21.2 (d, *J* = 478.8 Hz). HRMS (ESI) m/z calculated for $C_{17}H_{20}O_2P^+$ $[M+H]^+$ 287.1196 found 287.1193.

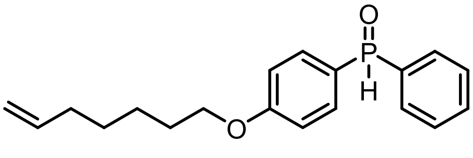

M7

According to **GP-Ⅱ**, **M7** was obtained as a colorless oil with a 31% overall yield. ^1H NMR (400 MHz, $CDCl_3$) δ 8.04 (d, *J* = 478.5 Hz, 1H), 7.75 – 7.45 (m, 7H), 6.97 (dq, *J* = 9.2, 2.6 Hz, 2H), 5.80 (ddt, *J* = 16.9, 10.2, 6.7 Hz, 1H), 5.05 – 4.90 (m, 2H), 3.98 (t, *J* = 6.5 Hz, 3H), 2.12 – 2.00 (m, 2H), 1.79 (ddt, *J* = 13.2, 9.9, 5.0 Hz, 2H), 1.51 – 1.40 (m, 4H). ^{13}C NMR (101 MHz, $CDCl_3$) δ 162.7, 138.8, 132.7 (d, *J* = 12.9 Hz), 132.5 (d, *J* = 2.9 Hz), 131.5, 130.8 (d, *J* = 11.4 Hz), 128.9 (d, *J* = 12.9 Hz), 122.3 (d, *J* = 107.9 Hz), 115.0 (d, *J* = 13.9 Hz), 114.6, 68.2, 33.7, 29.0, 28.7, 25.5. ^{31}P NMR (162 MHz, $CDCl_3$) δ 21.2 (d, *J* = 478.8 Hz). HRMS (ESI) m/z calculated for $C_{19}H_{24}O_2P^+$ $[M+H]^+$ 315.1509 found 315.1508.

The synthesis of **M8a**:

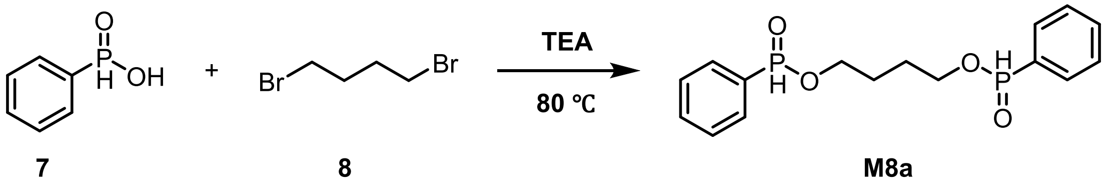

7 **8** **M8a**

To the **7** (0.63 g, 4.4 mmol) was added **8** (0.43g, 2 mmol) and Et_3N (0.45 g, 4.4 mmol), and the mixture was closed and heated at 80 ˚C for 3 h. Upon completion, the reaction was quenched with water. The mixture was extracted with ethyl acetate (EA), and the combined organic extracts were washed with brine, dried over anhydrous Na_2SO_4, and concentrated under reduced pressure. The residue was purified by flash chromatography on silica gel (DCM /MeOH=30:1) to afford **M8a** as a colorless oil (0.52 g, 77% yield). ^1H NMR (400 MHz, $CDCl_3$) δ 7.76 (ddq, J = 13.9, 6.9, 1.5 Hz, 4H), 7.57 (dd, J = 564.7, 2.6 Hz, 2H), 7.63 – 7.46 (m, 6H), 4.20 – 4.01 (m, 4H), 1.91 – 1.77 (m, 4H). ^{13}C NMR (101 MHz, $CDCl_3$) δ 133.3 (d, J = 3.0 Hz), 131.0 (d, J = 11.9 Hz), 129.7 (d, J = 132.2 Hz), 128.9 (d, J = 13.8 Hz), 65.2 (d, J = 6.5 Hz), 26.9 (d, J = 6.6 Hz). ^{31}P NMR (162 MHz, $CDCl_3$) δ 25.2 (d, J = 564.2 Hz). HRMS (ESI) m/z calculated for $C_{16}H_{21}O_4P_2^+$ $[M+H]^+$ 339.0910 found 339.0904.

The synthesis of **M11b**:

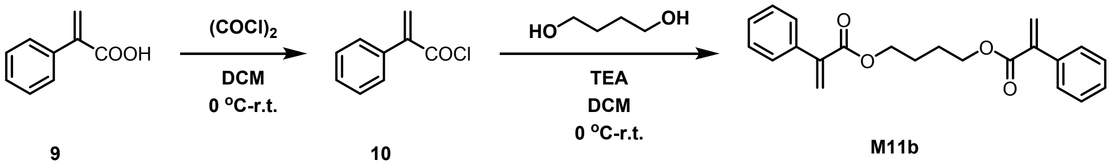

To a solution of the **9** (1.48 g, 10.0 mmol) in dry DCM (50 mL) at 0 ˚C under N_2 was added dropwise oxalyl chloride (2.54 g, 20.0 mmol) followed by a catalytic amount of dry DMF. The reaction mixture was stirred at room temperature 3 hours. The solvent were evaporated under reduced pressure and the resulting crude acid chloride was used directly for the next reaction without further purification. To a solution of 1,4-butanediol (0.45 g, 5.0 mmol) and Et_3N (1.51 g, 15 mmol) in DCM (50 mL) at 0 ˚C was added dropwise a solution of acyl chloride in DCM, the resulting mixture was stirred at room temperature for 3 hours. The mixture was diluted with DCM (30.0 mL) and washed with saturated $NaHCO_3$ (aq. 50 mL) and brine (50.0 mL) sequentially. The combined organic extracts were washed with brine, dried over anhydrous Na_2SO_4, and concentrated under reduced pressure. The residue was purified by flash chromatography on silica gel (DCM /MeOH=30:1) to afford **M11b** as a white solid (0.94 g, 27% yield). ^1H NMR (400 MHz, $CDCl_3$) δ 7.45 – 7.31 (m, 10H), 6.35 (d, J = 1.2 Hz, 2H), 5.90 (d, J = 1.3 Hz, 2H), 4.33 – 4.22 (m, 4H), 1.80 (h, J = 3.1 Hz, 4H). ^{13}C NMR (101 MHz, $CDCl_3$) δ 166.9, 141.5, 136.8, 128.4, 128.3, 128.2, 127.0, 64.7, 25.5. HRMS (ESI) m/z calculated for $C_{22}H_{26}NO_2^+$ $[M+NH_4]^+$ 368.1857 found 368.1852.

General Procedure for Polymerization:

All polymerization reactions were performed under a nitrogen atmosphere using a

standard Schlenk technique. The stock solution of DMPA was prepared in degassed DMF at a concentration of 100 mg/mL. The reactions were conducted in DMF under 365 nm UV irradiation at room temperature. After reaction, the mixture was diluted with DCM and precipitated into diethyl ether. A typical procedure for the preparation of **P1** was given below as an example. A 10 mL Schlenk vial equipped with a stir bar was charged with **M1** (1 mmol), followed by the stock solution of 100 mg/mL DMPA (128 μL), DMF (122 μL). After the vial was sealed, the mixture was deoxygenated via three freeze-pump-thaw cycles, backfilled with nitrogen, and then irradiated with a 24 W UV LED at room temperature for 16h. Then, the vial was opened to air to quench the polymerization. The crude product was diluted with DCM (0.3 mL) and precipitated into diethyl ether, yielding the polymer which was then characterized characterized by SEC, ^{1}H NMR, and ^{13}C NMR.

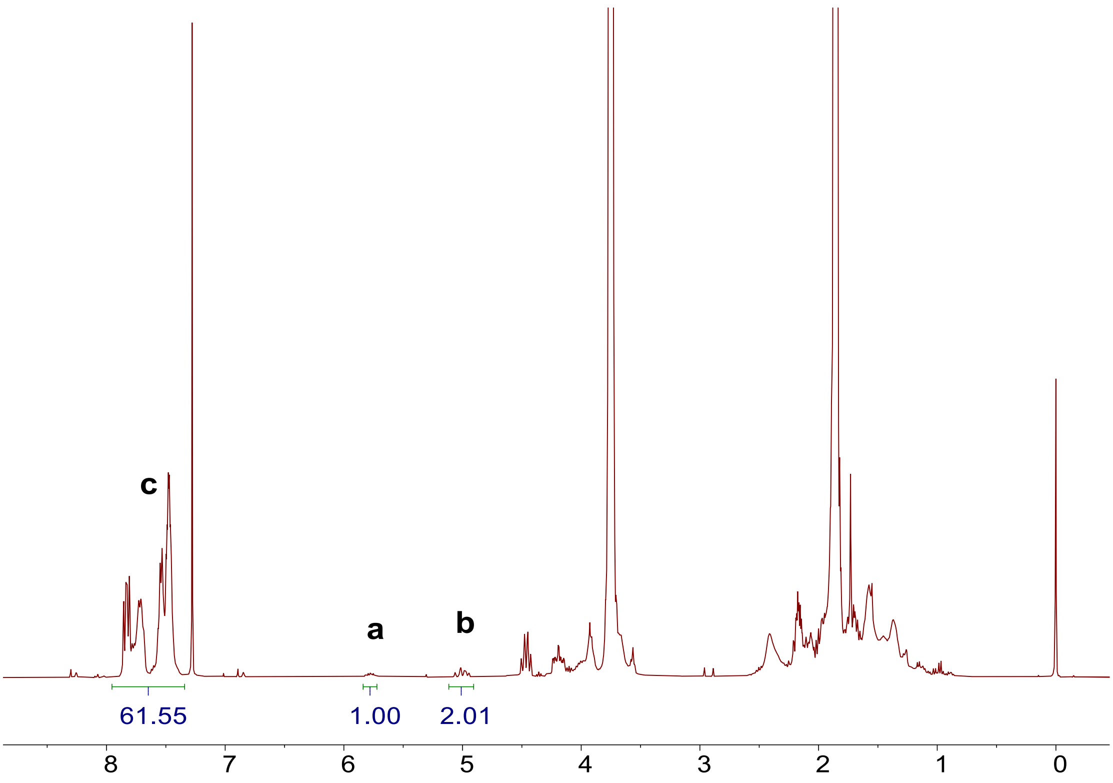

The peak at δ 5.83-5.72 (Peak a) is assigned to an atom on **M1** (vinyl H). The peak at δ 7.95-7.35 (Peak c) is assigned to aromatic protons. **M1** has five aromatic protons. When the integral of Peak a is normalized to 1, the conversion of **M1** α was determined based on the following equation S1-S2：

$$\alpha=\frac{c-5a}{c} \quad S1$$

$$\alpha=\frac{61.55-5*1}{61.55} = 92\%\ S2$$

General Procedure for kinetic experiment:

A 10 mL Schlenk vial equipped with a stir bar was charged with **M1** (2.64 mmol), followed by the stock solution of 100 mg/mL DMPA (338μL), DMF (322 μL). After the vial was sealed, the mixture was deoxygenated via three freeze-pump-thaw cycles, backfilled with nitrogen, and then irradiated with a 24 W UV LED at room temperature. 50 μL mixture as sample was extracted from the Schlenk vial with syringe under N_2 at various time interval. Then, the sample was opened to air, diluted with DCM, calculated conversion by ^{1}H NMR and precipitated in diethyl ether, yielding the polymer which was then characterized by SEC.

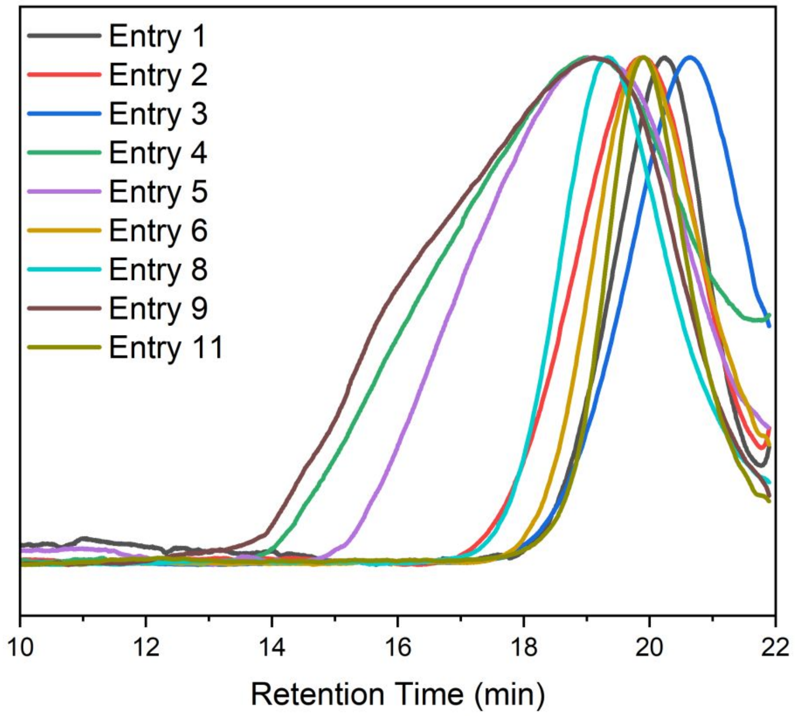

Figure S1. SEC trace of entries in Table 1.

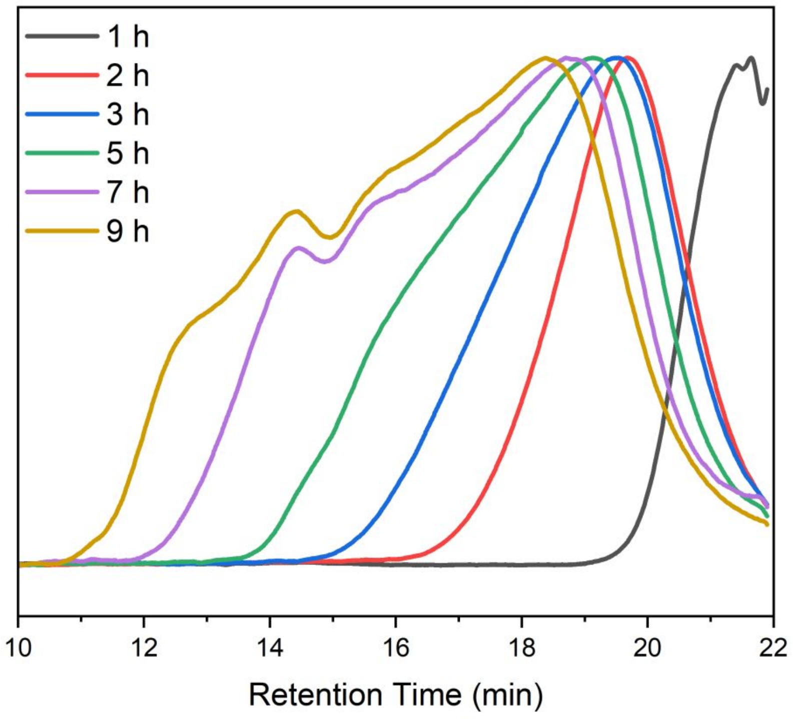

Figure S2. SEC trace from kinetic studies of **P1**.

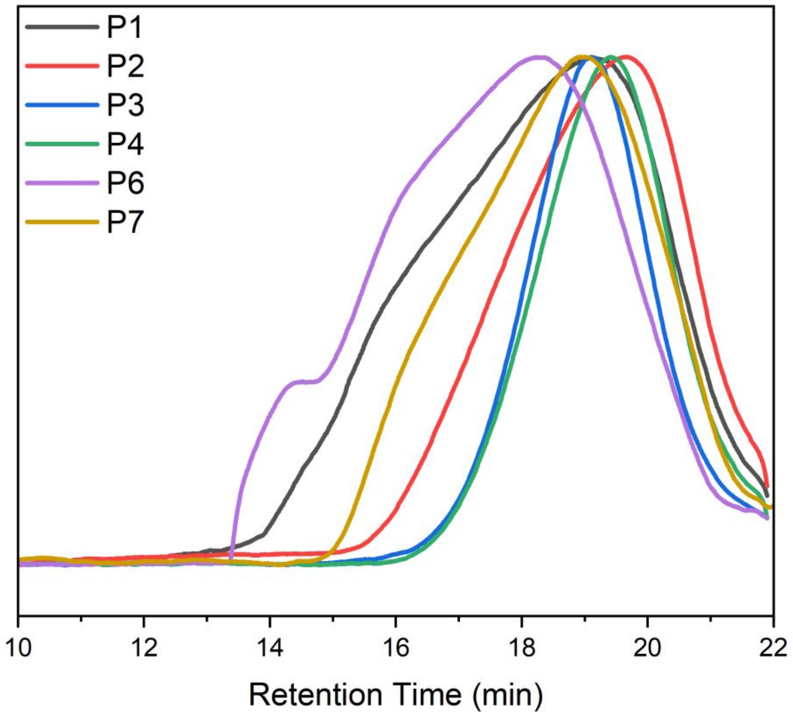

Figure S3. SEC trace of **P1-P7**.

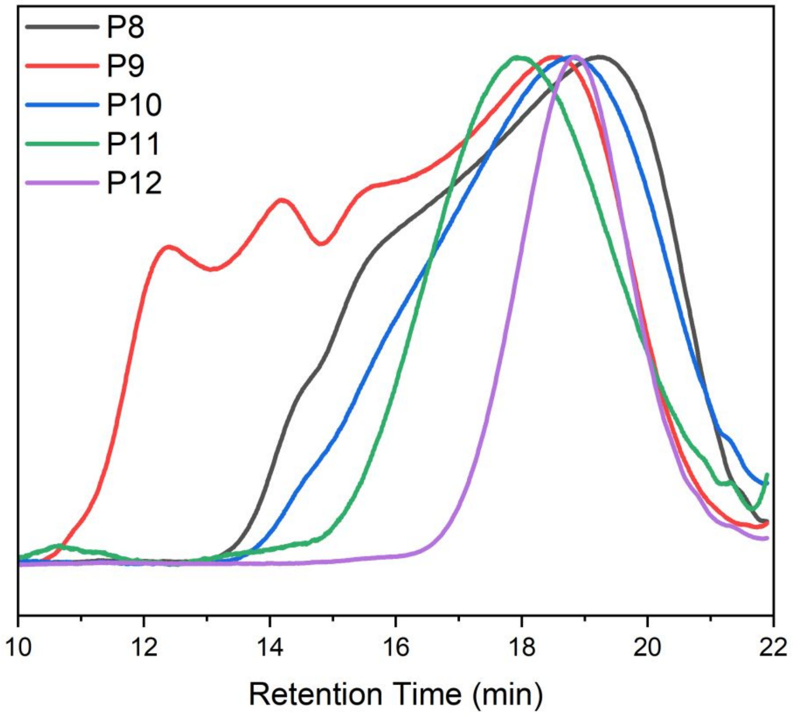

Figure S4. SEC trace of **P8-P12**.

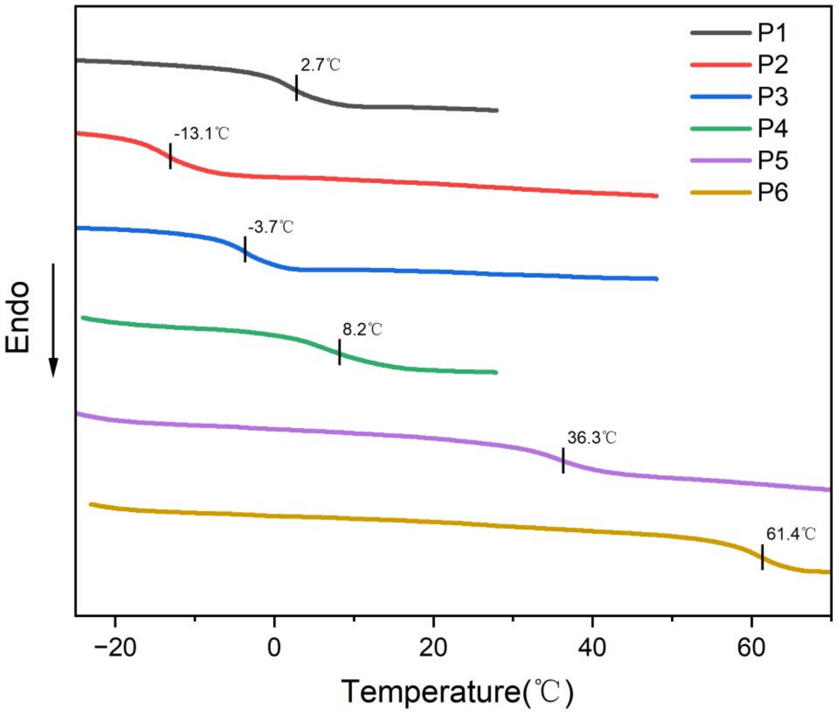

Figure S5. DSC thermograms of **P1-P6** were recorded under nitrogen during the second heating cycle at a heating rate of 10 ˚C /min.

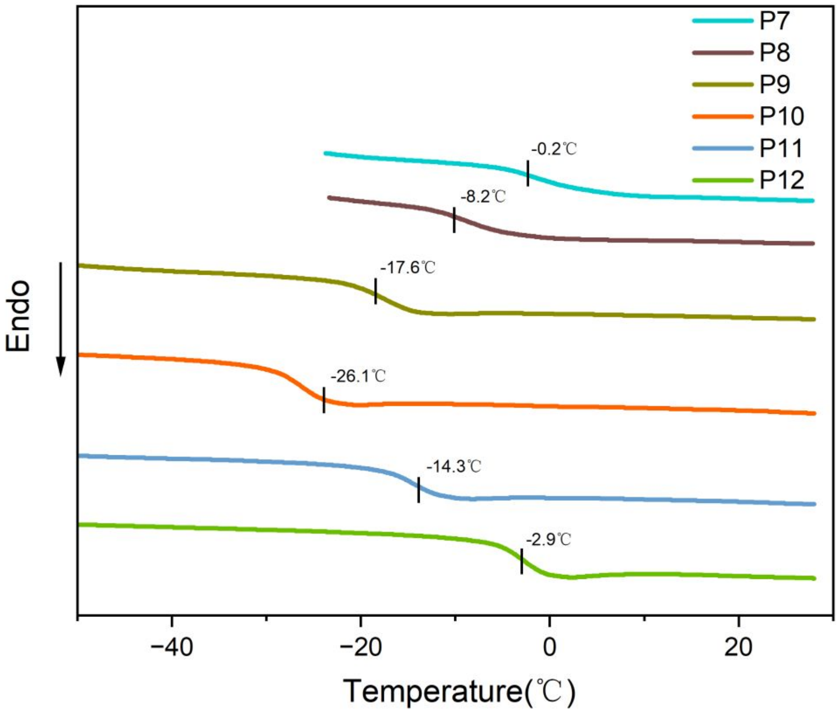

Figure S6. DSC thermograms of **P7-P12** were recorded under nitrogen during the second heating cycle at a heating rate of 10 ˚C /min.

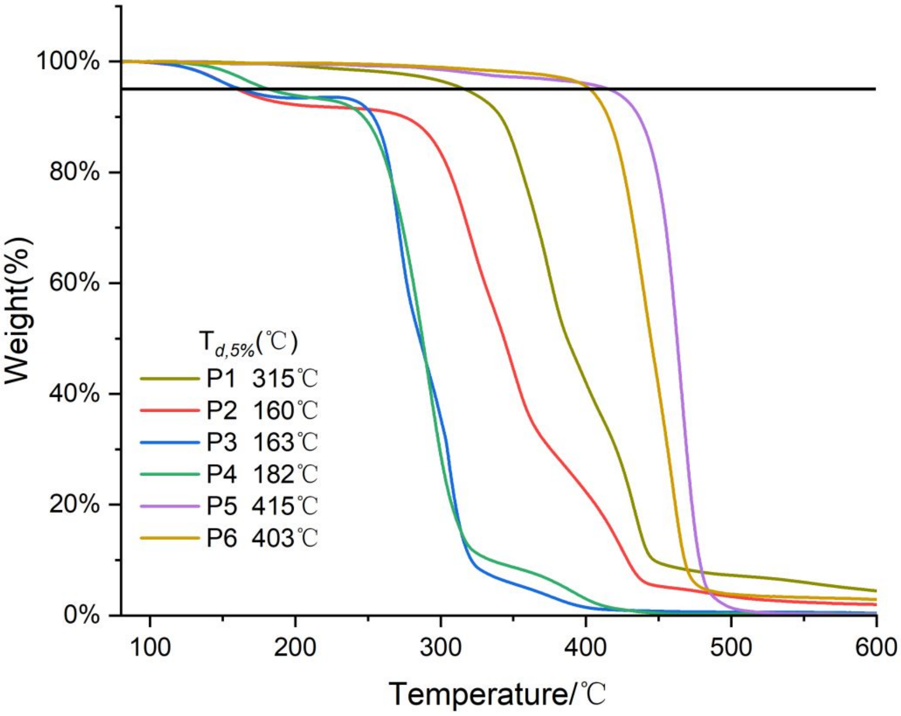

Figure S7. TGA analysis of **P1-P6**

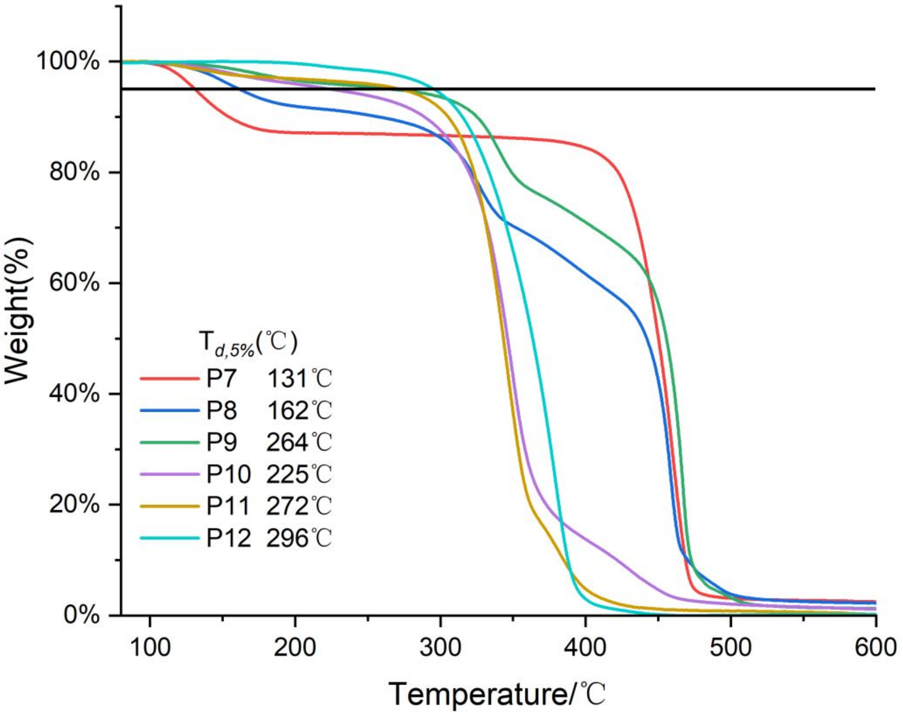

Figure S8. TGA analysis of **P7-P12**

Reference

(1) Kang, J.; Ding, K.; Ren, S.-M.; Yang, W.-J.; Su, B. Copper-Catalyzed Enantioselective Hydrophosphorylation of Unactivated Alkynes. *Angew. Chem. Int. Ed.* **2025**, *64*, e202415314.

(2) Huang, Y.-L.; Zhang, Q.-Q.; Wang, C.-Y.; Zhao, Y.; Wang, X.-S. Development of SF6 as a Formal Electrophilic Fluorinating Reagent for Photocatalyzed Oxidative Fluorination of Phosphine Oxides. *Org. Lett.* **2024**, *26*, 5776-5781.

(3) Shen, R.; Zhang, M.; Xiao, J.; Dong, C.; Han, L.-B. Ph3P-mediated highly selective C(α)–P coupling of quinone monoacetals with R2P(O)H: convenient and practical synthesis of ortho-phosphinyl phenols. *Green Chem.* **2018**, *20*, 5111-5116.

(4) Xu, Z.; Fang, N.; Zhao, Y. Calix[4]trap: A Bioinspired Host Equipped with Dual Selection Mechanisms. *J. Am. Chem. Soc.* **2021**, *143*, 3162-3168.

NMR Spectra

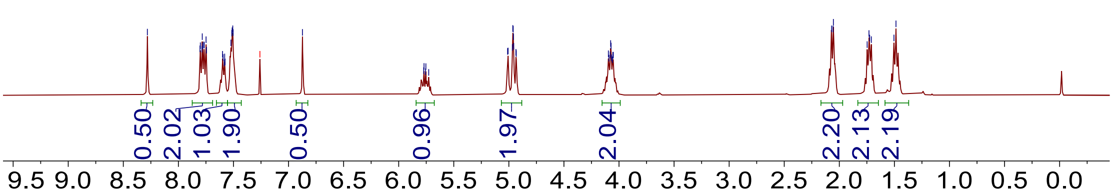

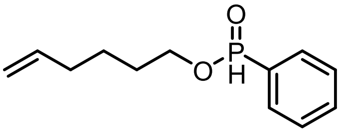

M1

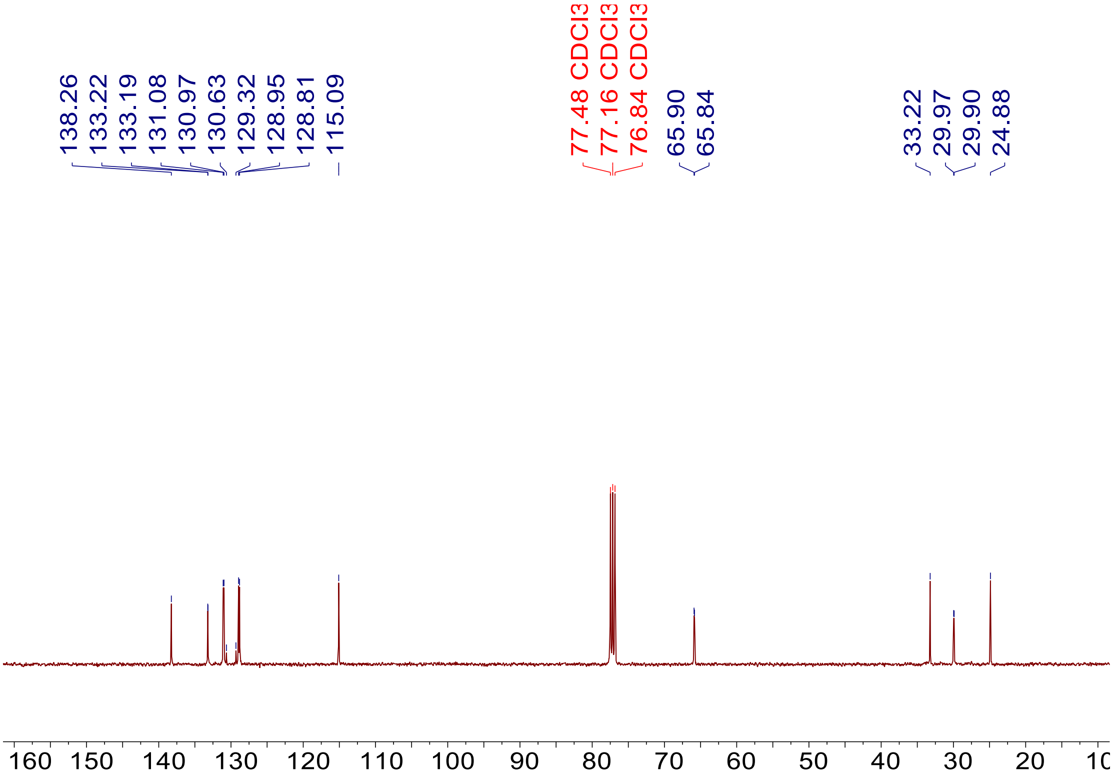

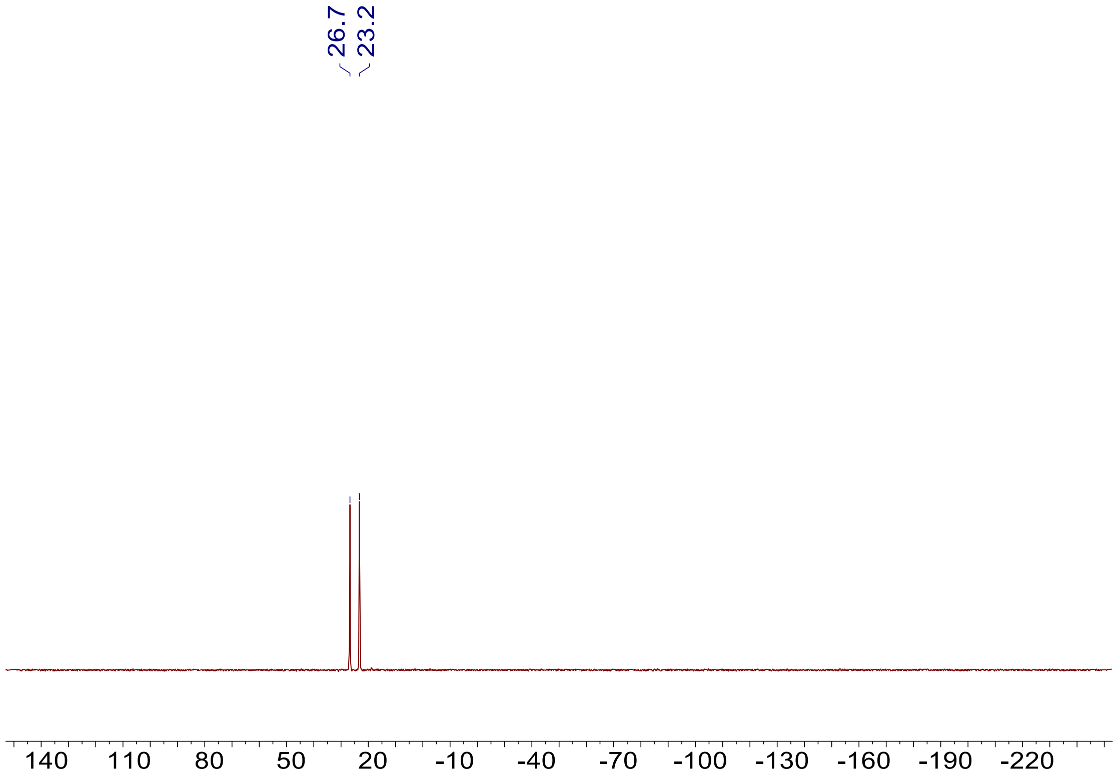
26.7
23.2
140 110 80 50 20 -10 -40 -70 -100 -130 -160 -190 -220

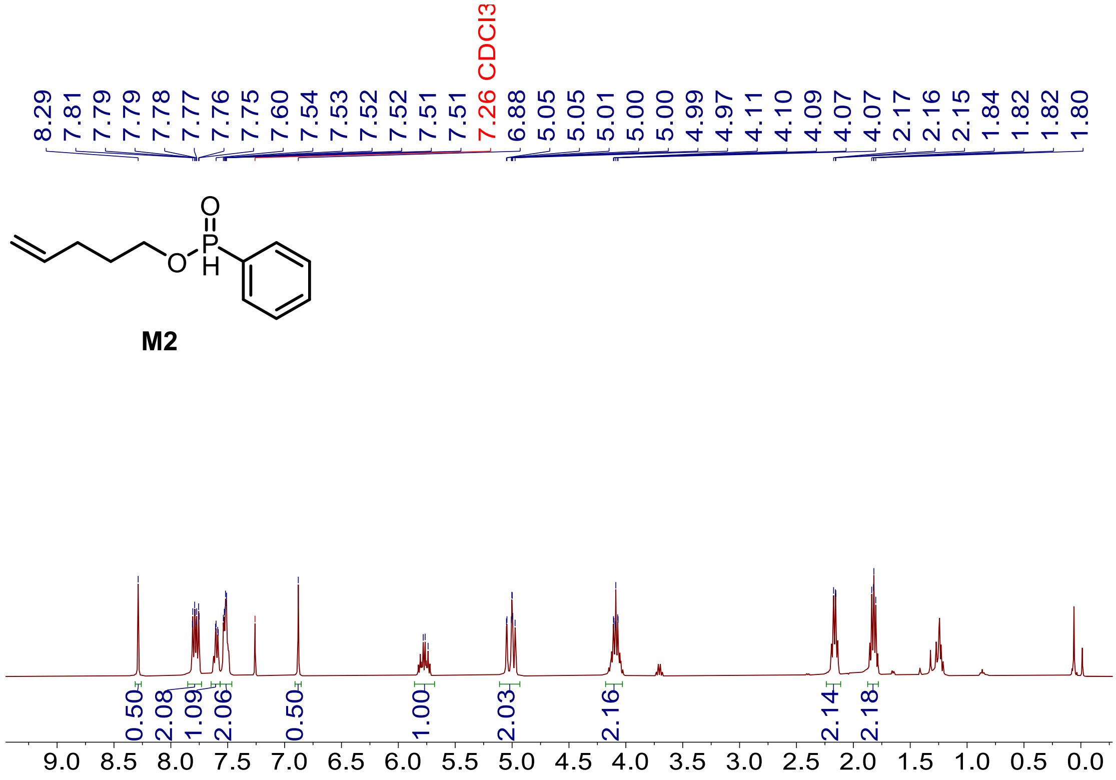
8.29 7.81 7.79 7.79 7.78 7.77 7.76 7.75 7.60 7.54 7.53 7.52 7.52 7.51 7.51
7.26 CDCl3
M2
6.88 5.05 5.05 5.01 5.00 5.00 4.99 4.97 4.11 4.10 4.09 4.07 4.07 2.17 2.16 2.15 1.84 1.82 1.82 1.80
0.50 2.08 1.09 2.06 0.50 1.00 2.03 2.16 2.14 2.18
9.0 8.5 8.0 7.5 7.0 6.5 6.0 5.5 5.0 4.5 4.0 3.5 3.0 2.5 2.0 1.5 1.0 0.5 0.0

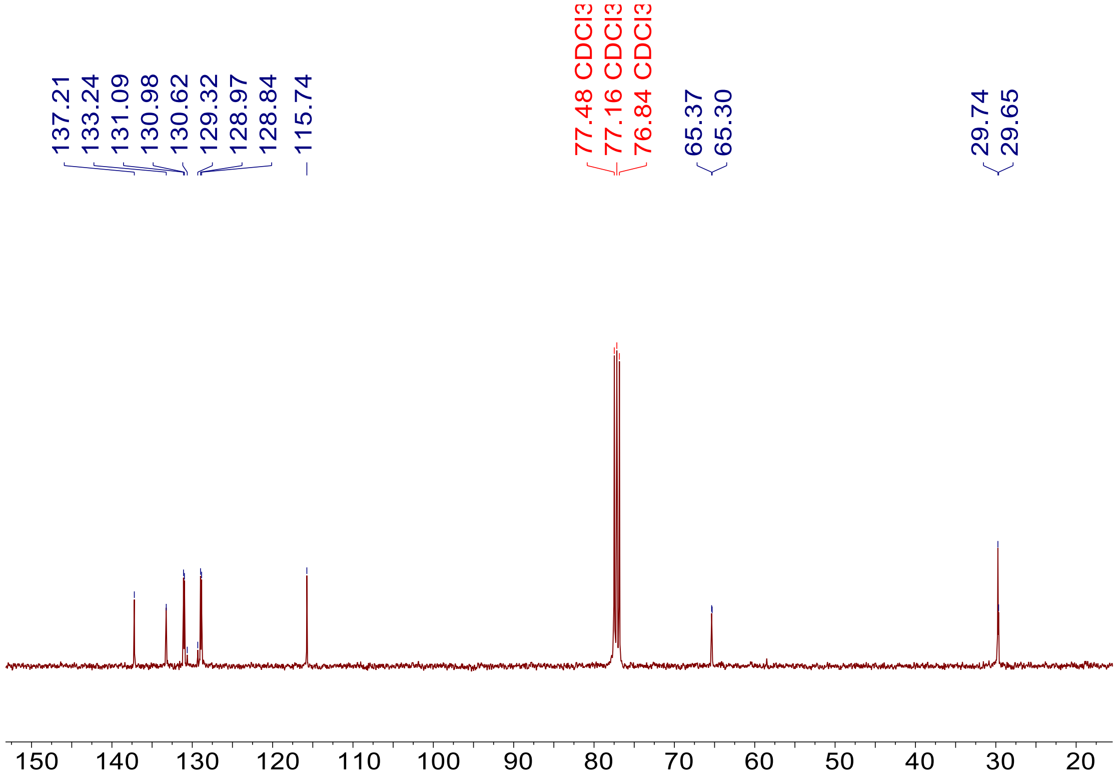
137.21
133.24
131.09
130.98
130.62
129.32
128.97
128.84
115.74
77.48 CDCl3
77.16 CDCl3
76.84 CDCl3
65.37
65.30
29.74
29.65
150 140 130 120 110 100 90 80 70 60 50 40 30 20

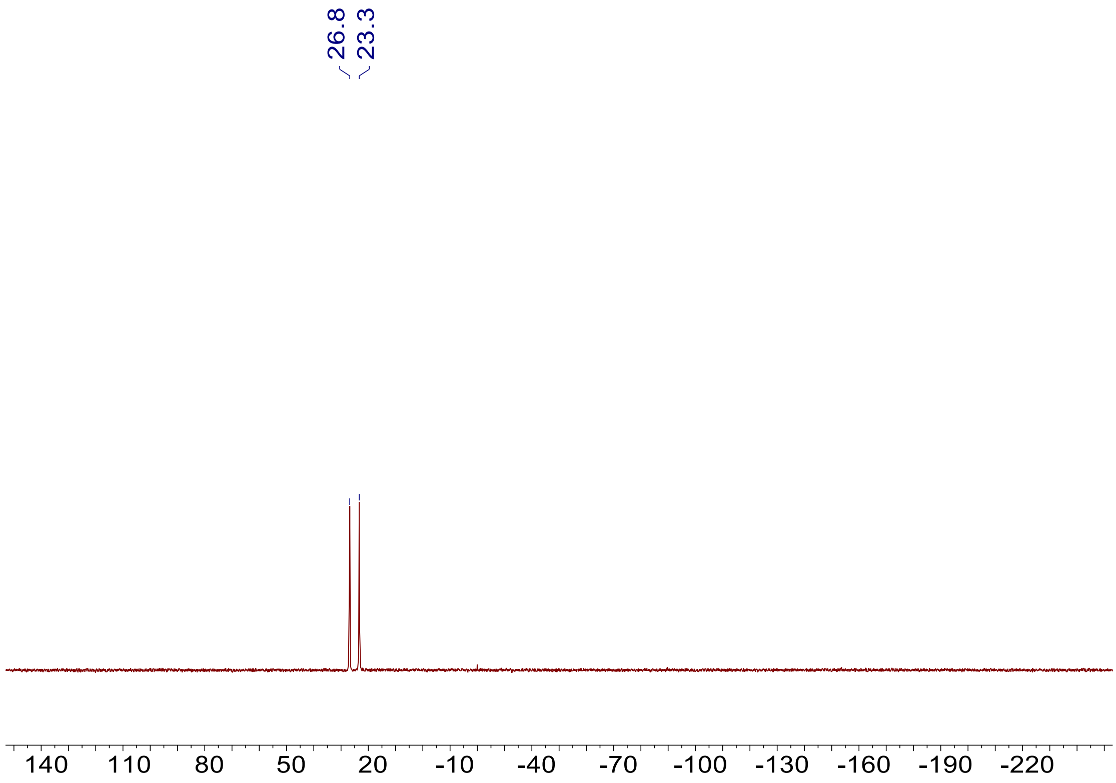
26.8
23.3
140 110 80 50 20 -10 -40 -70 -100 -130 -160 -190 -220

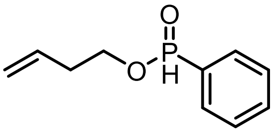

M3

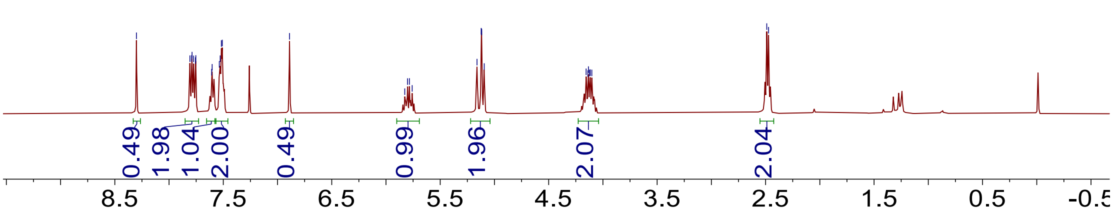
8.30
7.81
7.79
7.79
7.77
7.76
7.75
7.61
7.60
7.54
7.53
7.52
7.51
6.89
5.83
5.80
5.78
5.76
5.16
5.12
5.12
5.09
4.15
4.14
4.13
4.12
4.12
4.10
2.49
2.47
0.49
1.98
1.04
2.00
0.49
0.99
1.96
2.07
2.04
8.5
7.5
6.5
5.5
4.5
3.5
2.5
1.5
0.5
-0.5

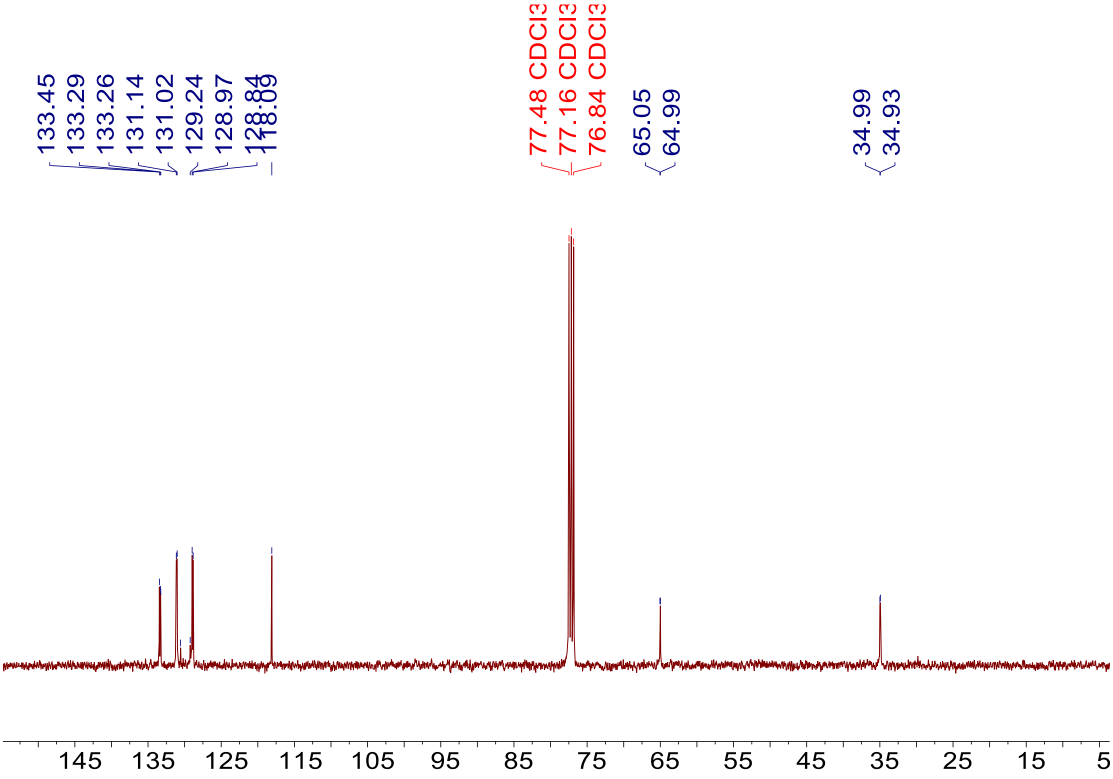
133.45
133.29
133.26
131.14
131.02
129.24
128.97
128.84
118.09
77.48 CDCl3
77.16 CDCl3
76.84 CDCl3
65.05
64.99
34.99
34.93
145
135
125
115
105
95
85
75
65
55
45
35
25
15
5

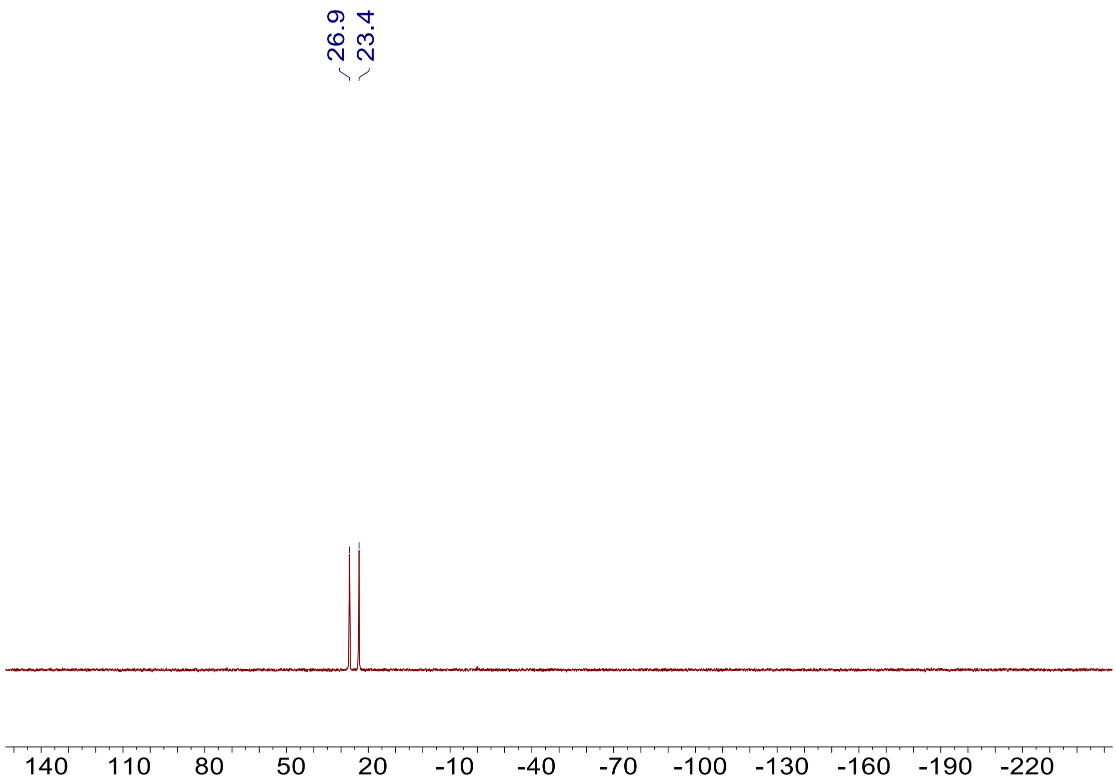
26.9
23.4
140
110
80
50
20
-10
-40
-70
-100
-130
-160
-190
-220

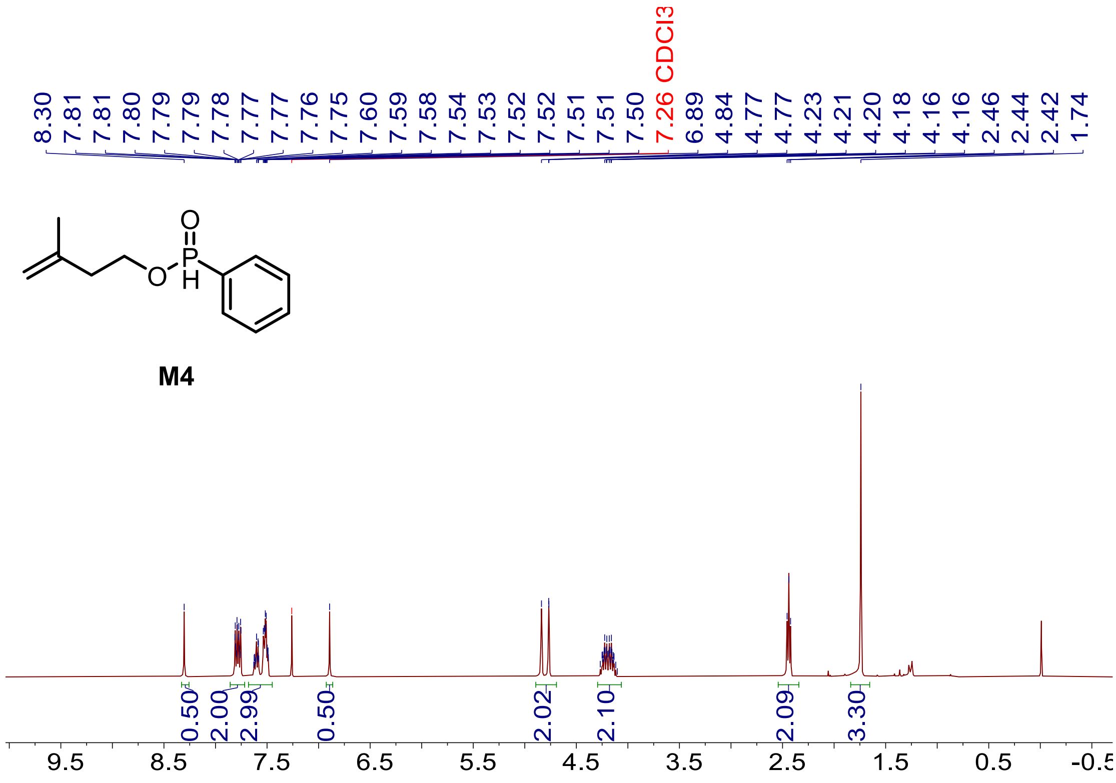
8.30
7.81
7.81
7.80
7.79
7.79
7.78
7.77
7.77
7.76
7.75
7.60
7.59
7.58
7.54
7.53
7.52
7.52
7.51
7.51
7.50
7.26 CDCl3
6.89
4.84
4.77
4.77
4.23
4.21
4.20
4.18
4.16
4.16
2.46
2.44
2.42
1.74
0.50
2.00
2.99
0.50
2.02
2.10
2.09
3.30
9.5
8.5
7.5
6.5
5.5
4.5
3.5
2.5
1.5
0.5
-0.5

M4

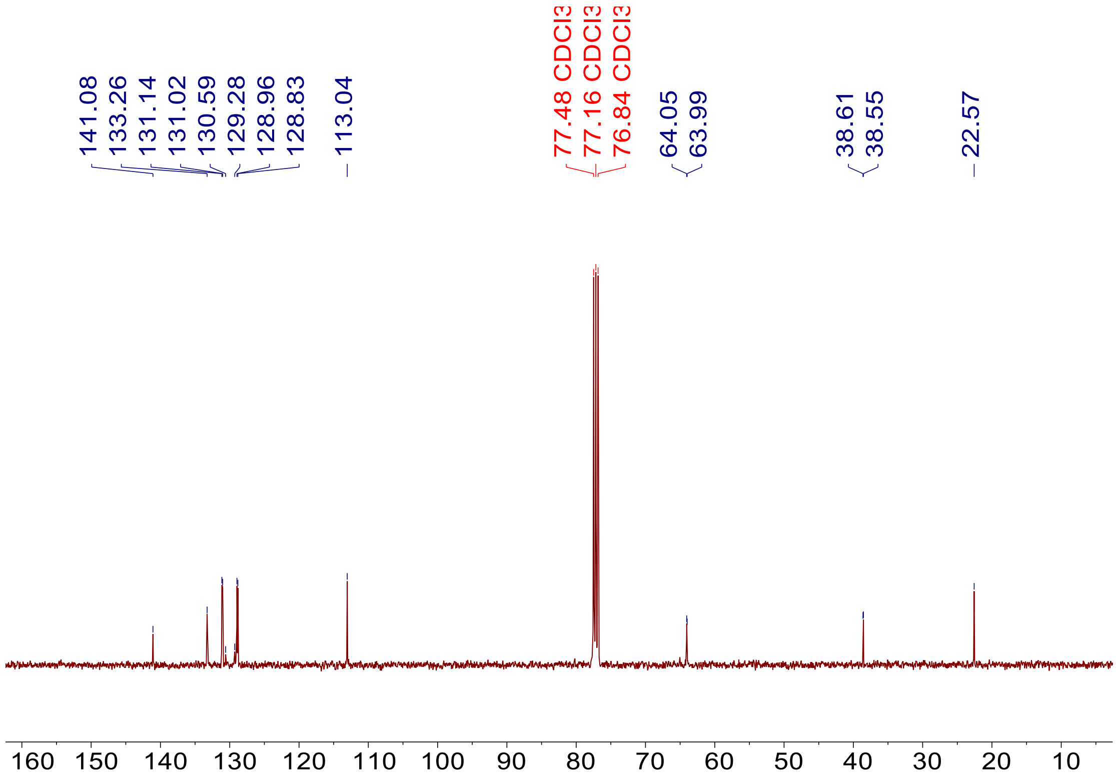
141.08
133.26
131.14
131.02
130.59
129.28
128.96
128.83
113.04
77.48 CDCl3
77.16 CDCl3
76.84 CDCl3
64.05
63.99
38.61
38.55
22.57
160 150 140 130 120 110 100 90 80 70 60 50 40 30 20 10

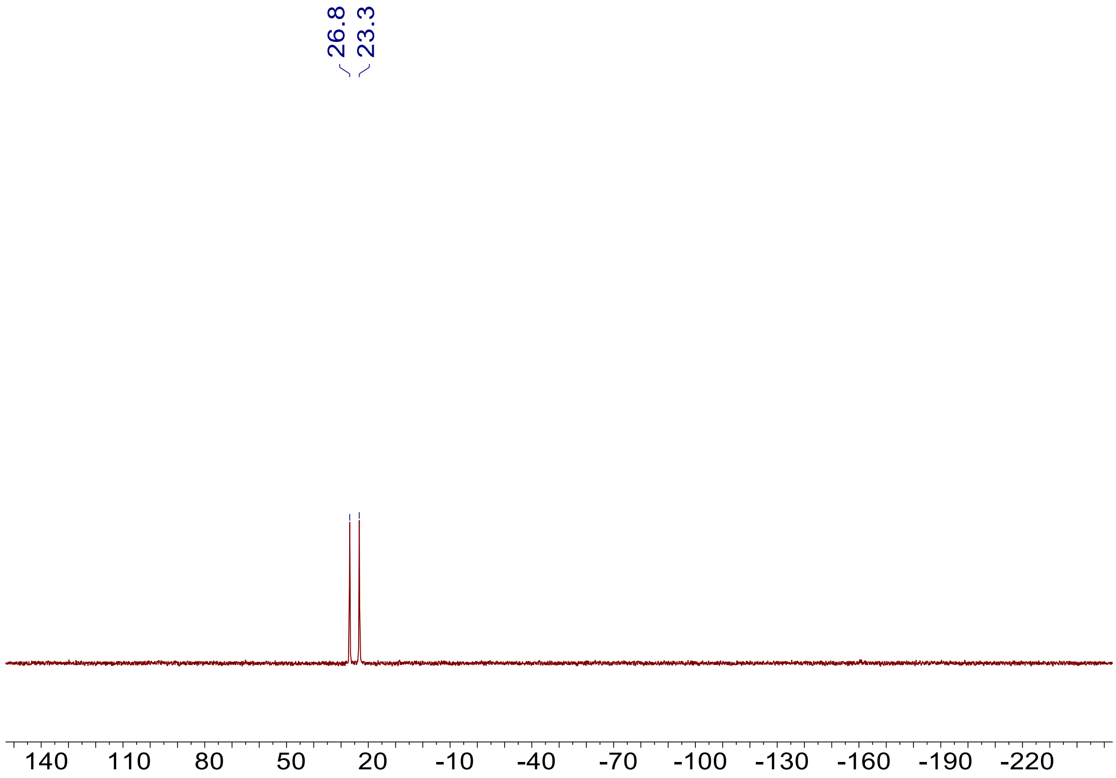
26.8
23.3
140 110 80 50 20 -10 -40 -70 -100 -130 -160 -190 -220

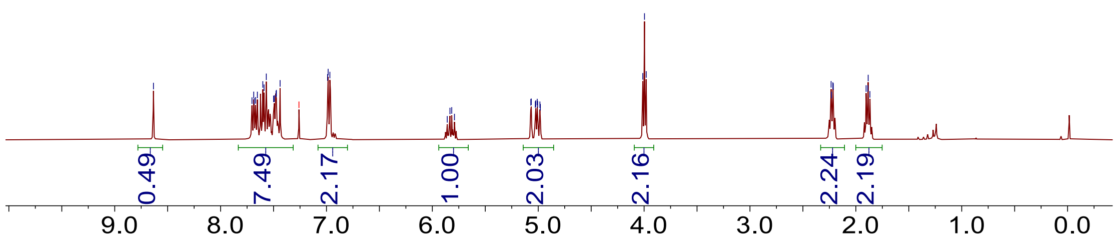
8.63
7.70
7.69
7.68
7.67
7.65
7.60
7.59
7.57
7.50
7.49
7.48
7.47
7.44
7.26 CDCl3
6.99
6.98
6.97
5.07
5.07
5.03
5.02
5.01
5.01
4.98
4.01
4.00
3.98
2.23
2.23
2.22
2.21
1.90
1.89
1.88
1.87
0.49
7.49
2.17
1.00
2.03
2.16
2.24
2.19
9.0
8.0
7.0
6.0
5.0
4.0
3.0
2.0
1.0
0.0

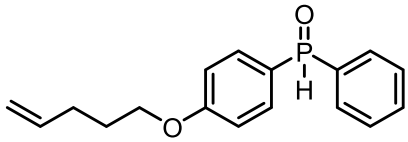
O
P
H
O

M6

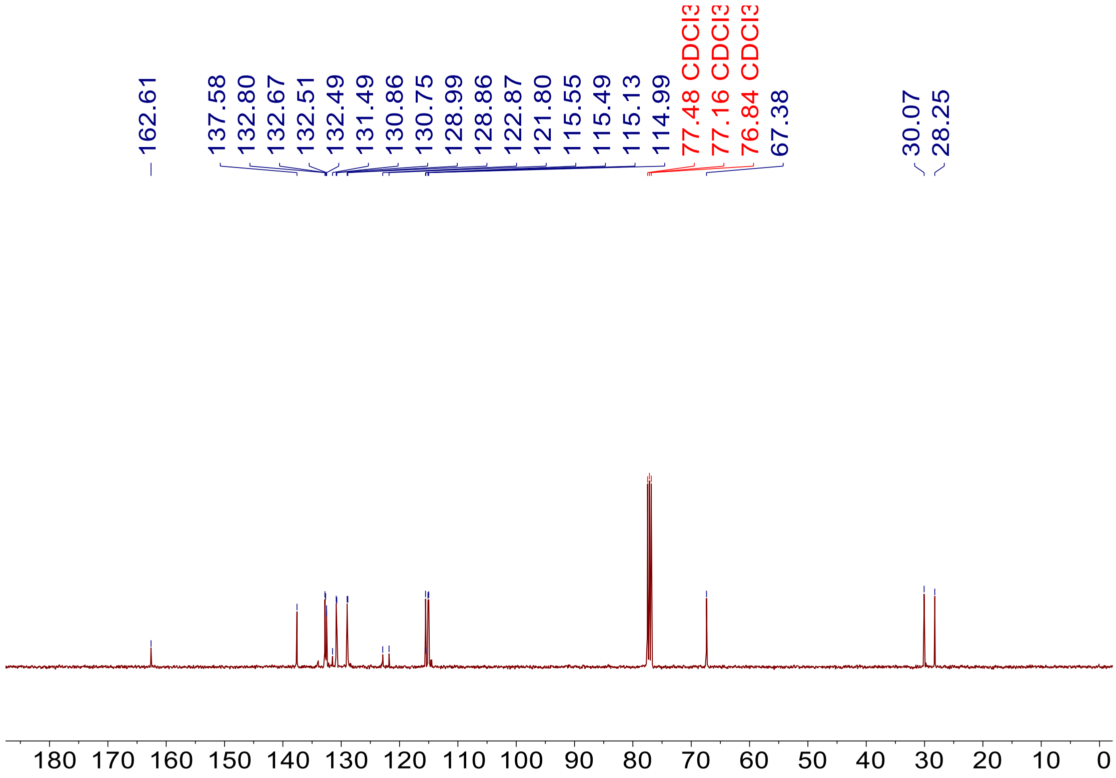
162.61
137.58
132.80
132.67
132.51
132.49
131.49
130.86
130.75
128.99
128.86
122.87
121.80
115.55
115.49
115.13
114.99
77.48 CDCl3
77.16 CDCl3
76.84 CDCl3
67.38
30.07
28.25
180
170
160
150
140
130
120
110
100
90
80
70
60
50
40
30
20
10
0

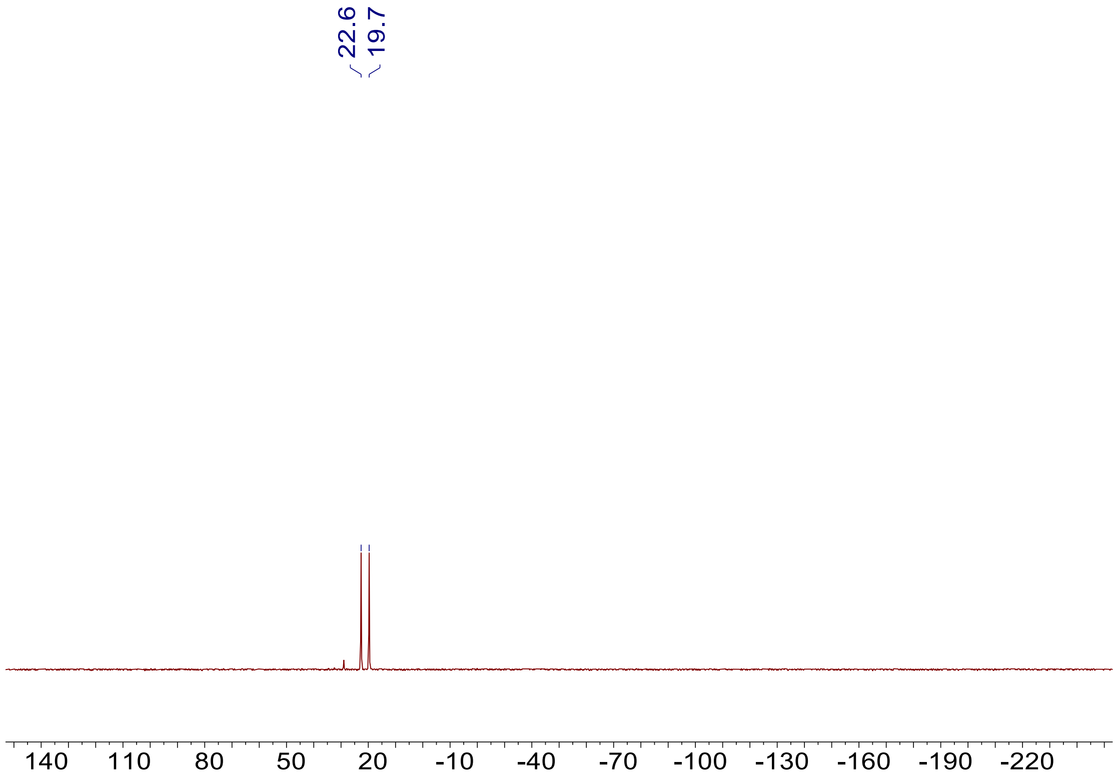
22.6
19.7
140 110 80 50 20 -10 -40 -70 -100 -130 -160 -190 -220

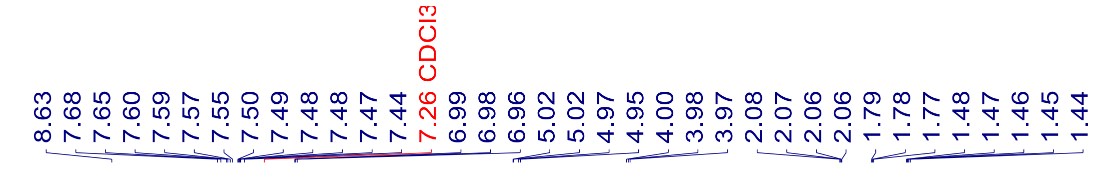
8.63 7.68 7.65 7.60 7.59 7.57 7.55 7.50 7.49 7.48 7.48 7.47 7.44 7.26 CDCl3 6.99 6.98 6.96 5.02 5.02 4.97 4.95 4.00 3.98 3.97 2.08 2.07 2.06 2.06 1.79 1.78 1.77 1.48 1.47 1.46 1.45 1.44

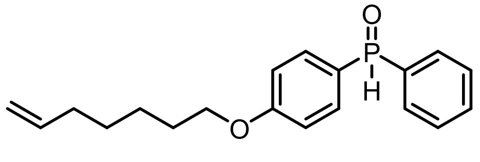
O
P
H
O

M7

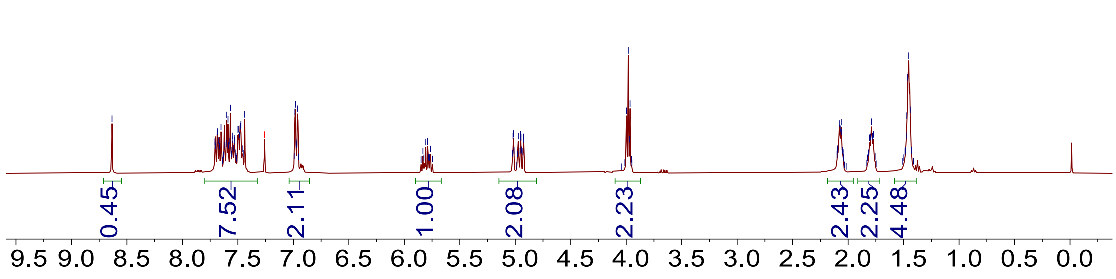
0.45 7.52 2.11 1.00 2.08 2.23 2.43 2.25 4.48
9.5 9.0 8.5 8.0 7.5 7.0 6.5 6.0 5.5 5.0 4.5 4.0 3.5 3.0 2.5 2.0 1.5 1.0 0.5 0.0

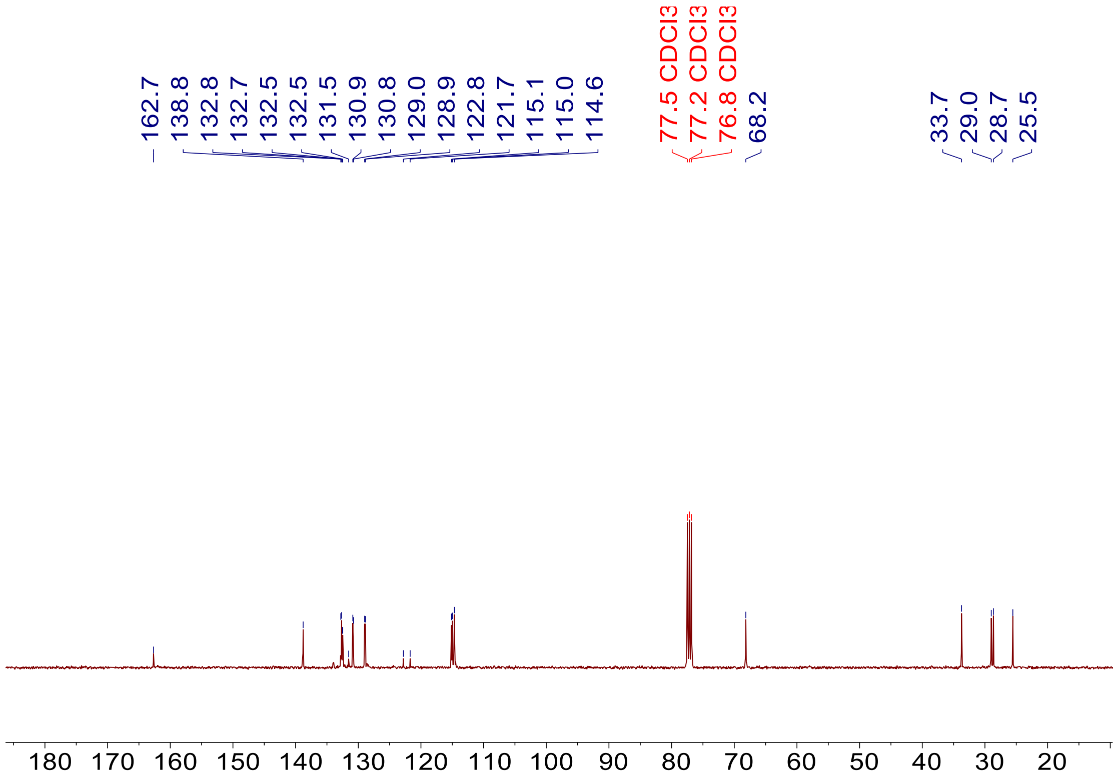
162.7
138.8
132.8
132.7
132.5
132.5
131.5
130.9
130.8
129.0
128.9
122.8
121.7
115.1
115.0
114.6
77.5 CDCl3
77.2 CDCl3
76.8 CDCl3
68.2
33.7
29.0
28.7
25.5
180 170 160 150 140 130 120 110 100 90 80 70 60 50 40 30 20

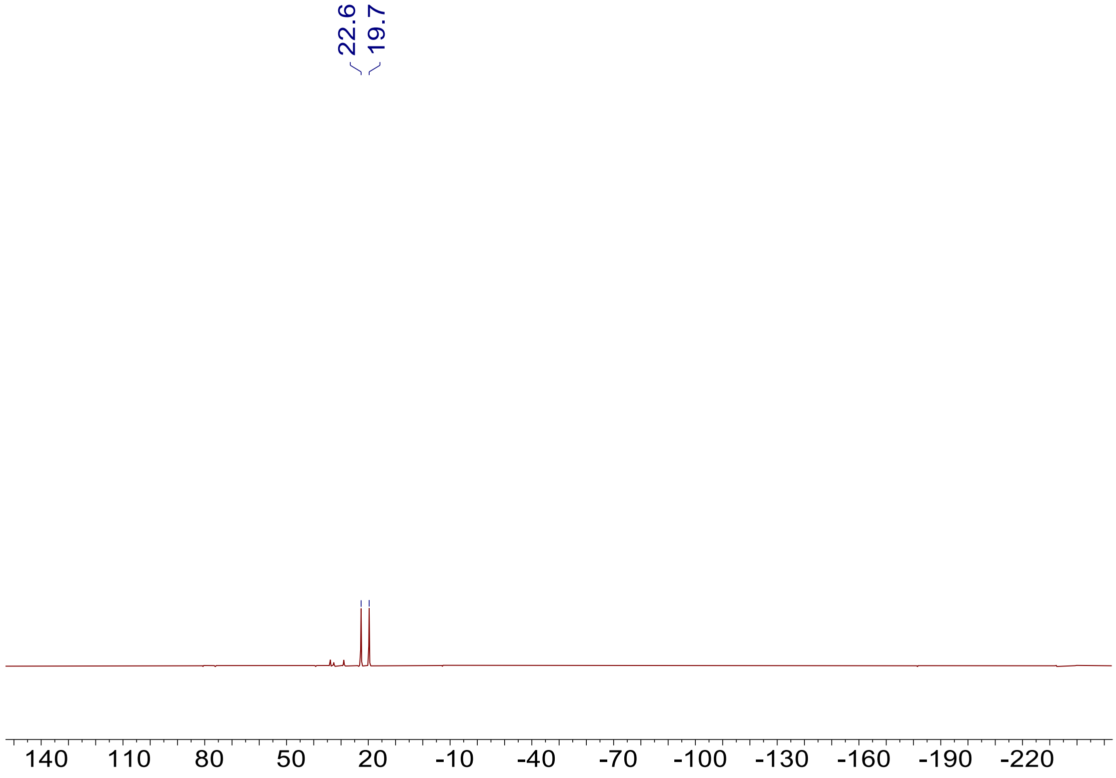
22.6
19.7
140 110 80 50 20 -10 -40 -70 -100 -130 -160 -190 -220

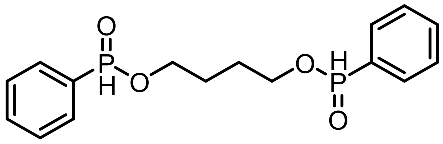

M8a

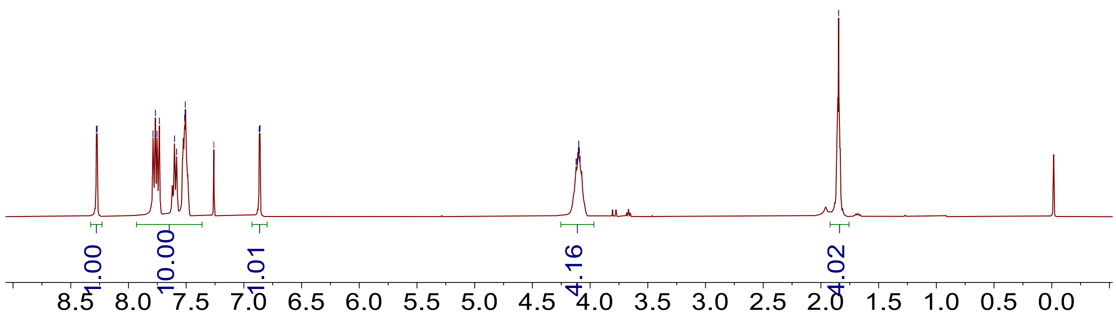
8.28
8.27
7.79
7.77
7.75
7.73
7.60
7.58
7.51
7.51
7.50
7.26 CDCl3
6.87
6.86
4.12
4.11
4.11
4.10
4.10
4.09
4.09
1.85
1.00
10.00
1.01
4.16
4.02
8.5 8.0 7.5 7.0 6.5 6.0 5.5 5.0 4.5 4.0 3.5 3.0 2.5 2.0 1.5 1.0 0.5 0.0

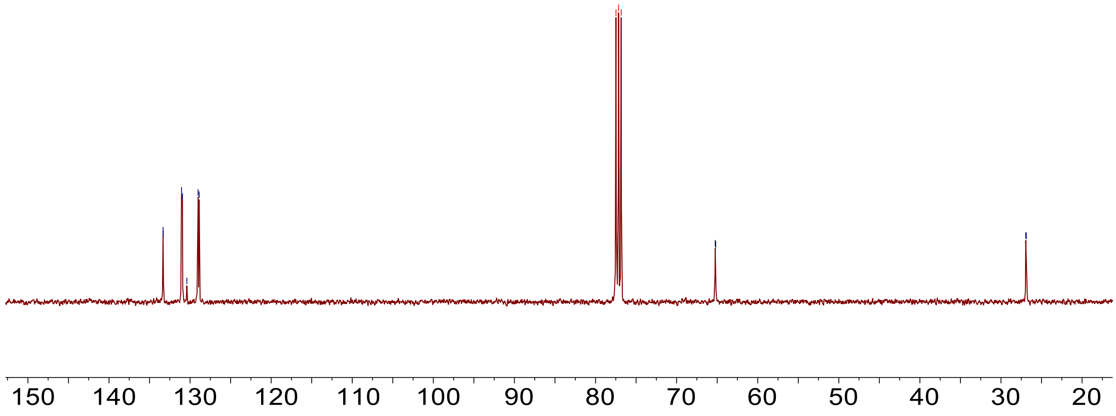
133.33
133.30
131.06
130.94
130.40
129.01
128.87
77.48 CDCl3
77.16 CDCl3
76.84 CDCl3
65.24
65.18
26.95
26.88
150 140 130 120 110 100 90 80 70 60 50 40 30 20

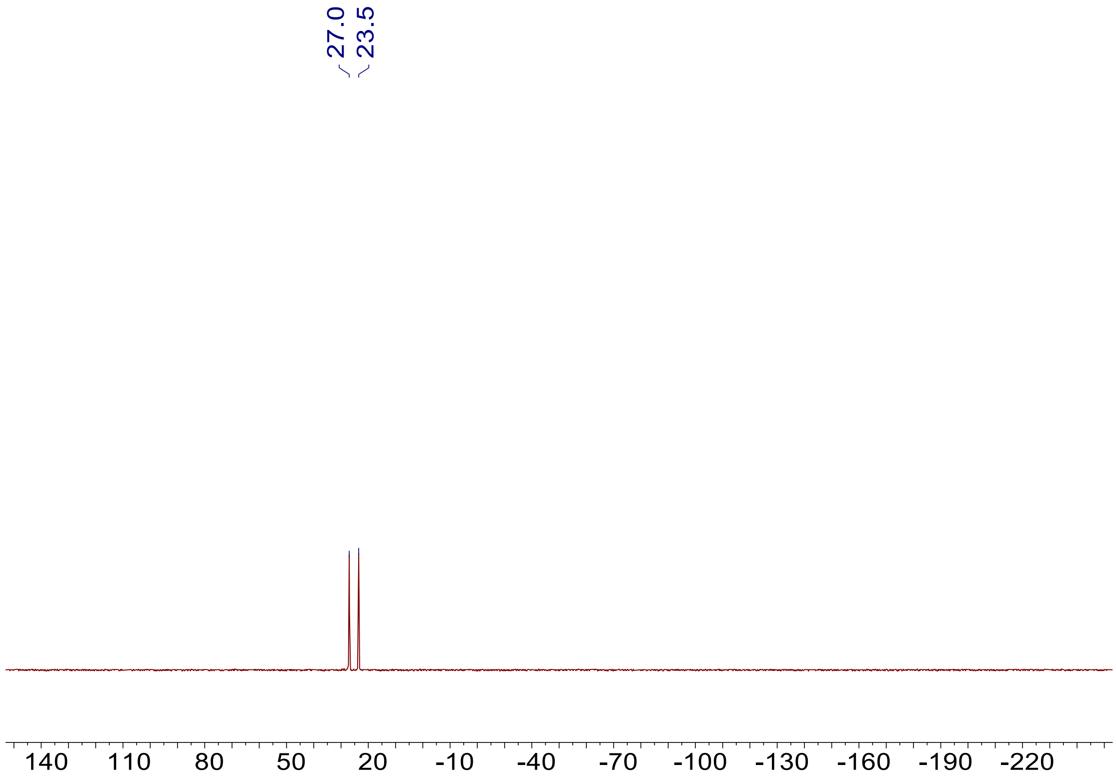
27.0
23.5
140 110 80 50 20 -10 -40 -70 -100 -130 -160 -190 -220

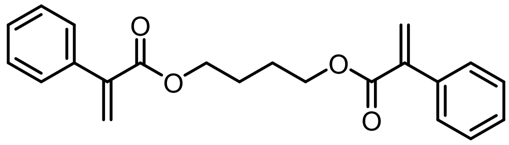
O
O
O
O

M11b

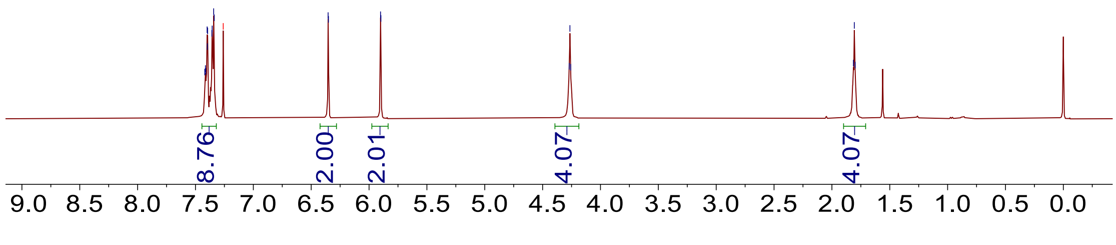
7.41 7.40 7.40 7.40 7.36 7.36 7.34 7.34
7.26 CDCl3
6.35 6.35 5.90 5.90
4.27 4.26 4.26
1.81 1.81 1.80
8.76 2.00 2.01 4.07 4.07
9.0 8.5 8.0 7.5 7.0 6.5 6.0 5.5 5.0 4.5 4.0 3.5 3.0 2.5 2.0 1.5 1.0 0.5 0.0

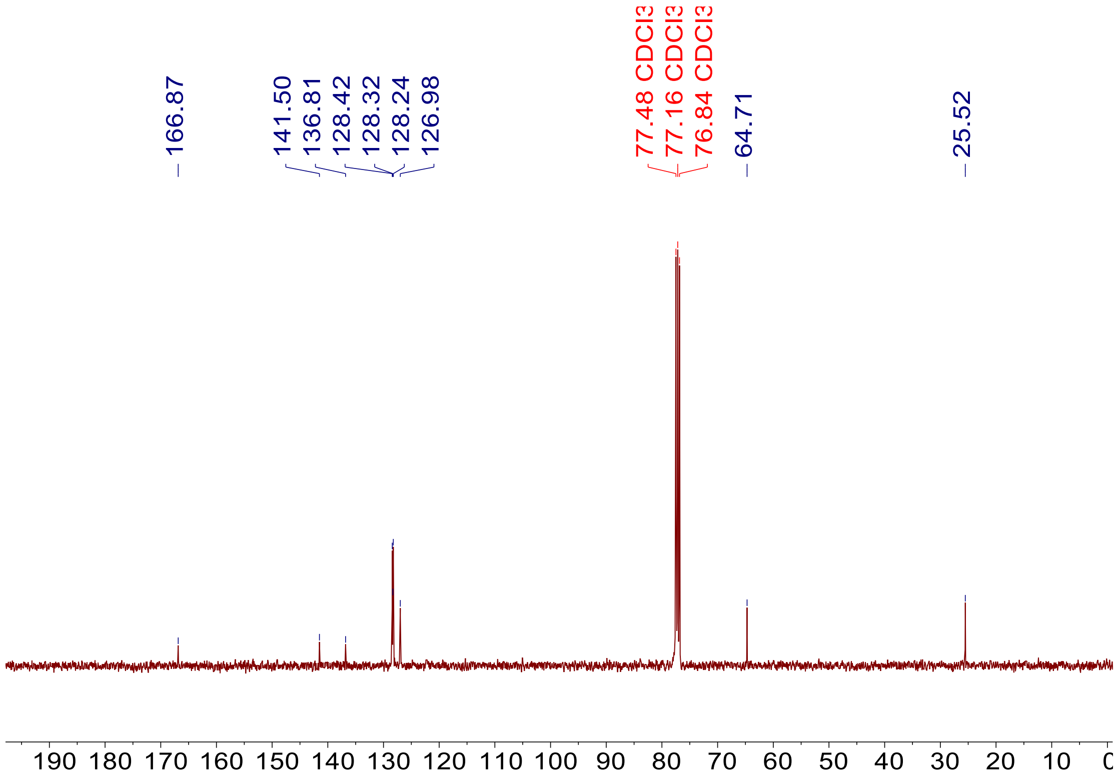
166.87
141.50
136.81
128.42
128.32
128.24
126.98
77.48 CDCl3
77.16 CDCl3
76.84 CDCl3
64.71
25.52
190 180 170 160 150 140 130 120 110 100 90 80 70 60 50 40 30 20 10

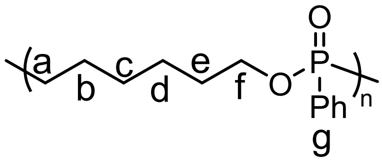
a
b
c
d
e
f
O
P
Ph
g
n

P1

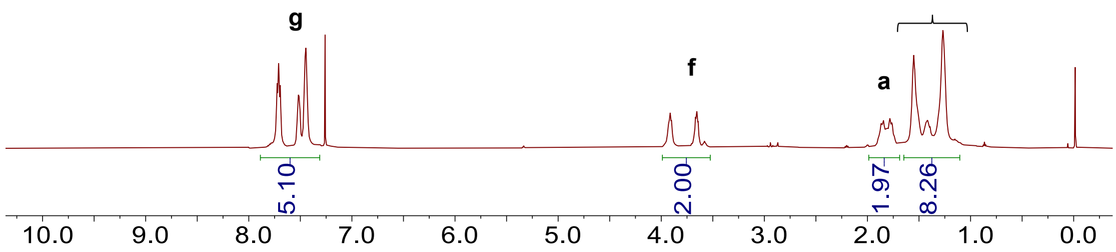
g
f
a
b, c, d, e
5.10
2.00
1.97
8.26
10.0 9.0 8.0 7.0 6.0 5.0 4.0 3.0 2.0 1.0 0.0

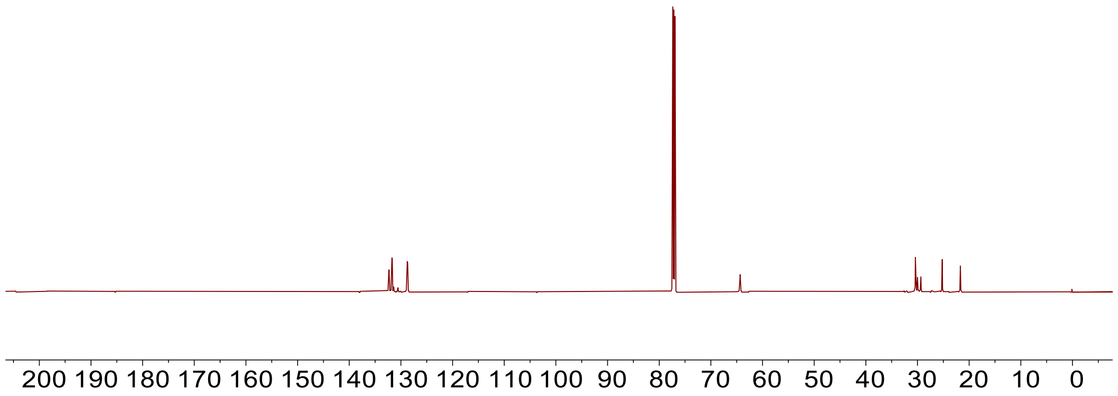
200 190 180 170 160 150 140 130 120 110 100 90 80 70 60 50 40 30 20 10 0

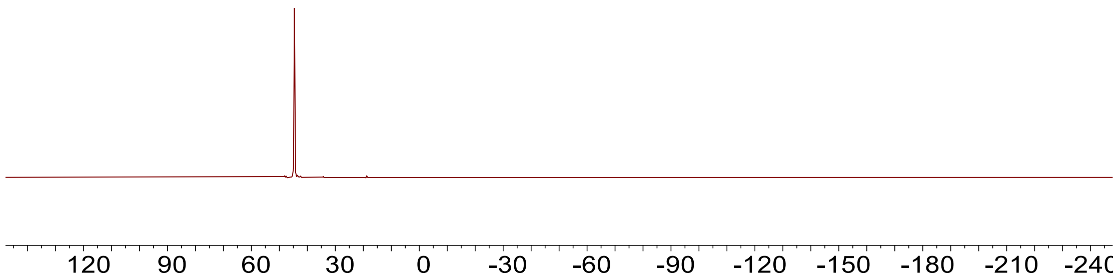
120 90 60 30 0 -30 -60 -90 -120 -150 -180 -210 -240

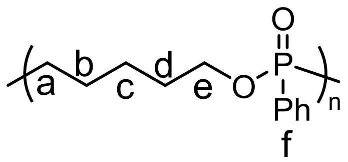
O
P
a
b
c
d
e
O
Ph
f
n

P2

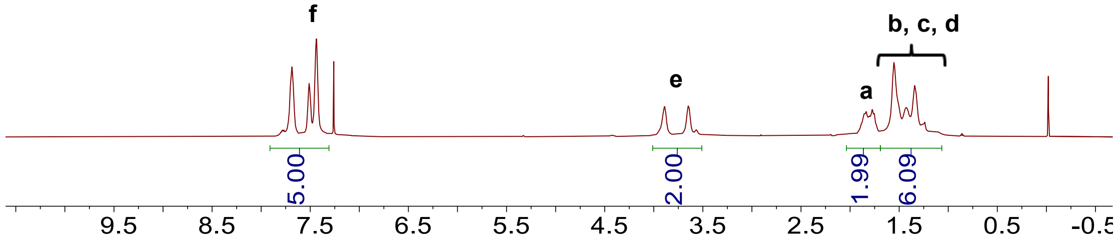
f
e
a
b, c, d
5.00
2.00
1.99
6.09
9.5
8.5
7.5
6.5
5.5
4.5
3.5
2.5
1.5
0.5
-0.5

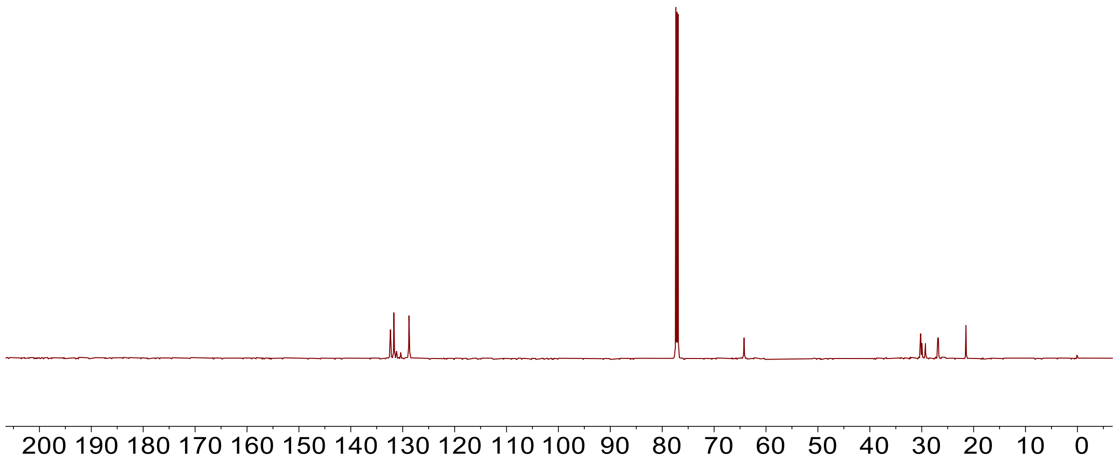
200 190 180 170 160 150 140 130 120 110 100 90 80 70 60 50 40 30 20 10 0

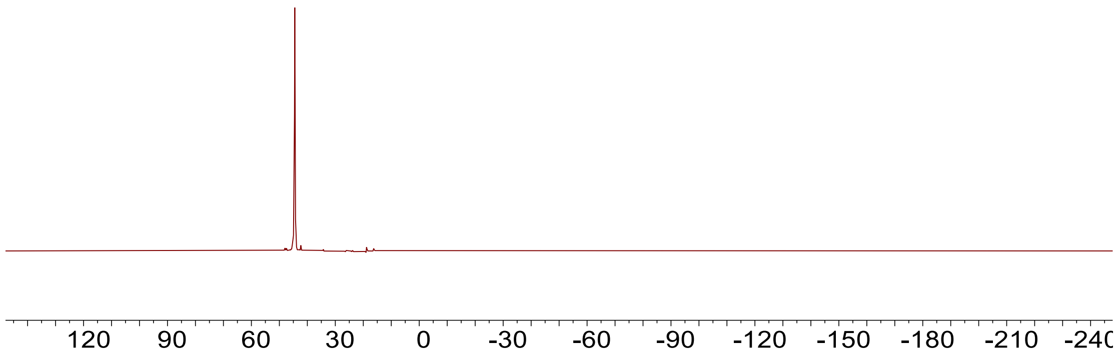
120
90
60
30
0
-30
-60
-90
-120
-150
-180
-210
-240

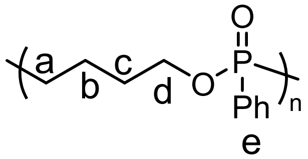
a
b
c
d
O
P
O
Ph
n
e

P3

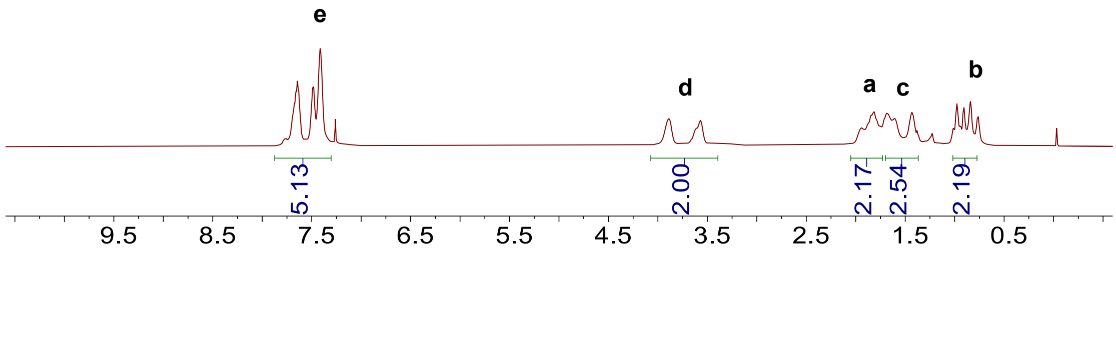
e
d
a
c
b
5.13
2.00
2.17
2.54
2.19
9.5
8.5
7.5
6.5
5.5
4.5
3.5
2.5
1.5
0.5

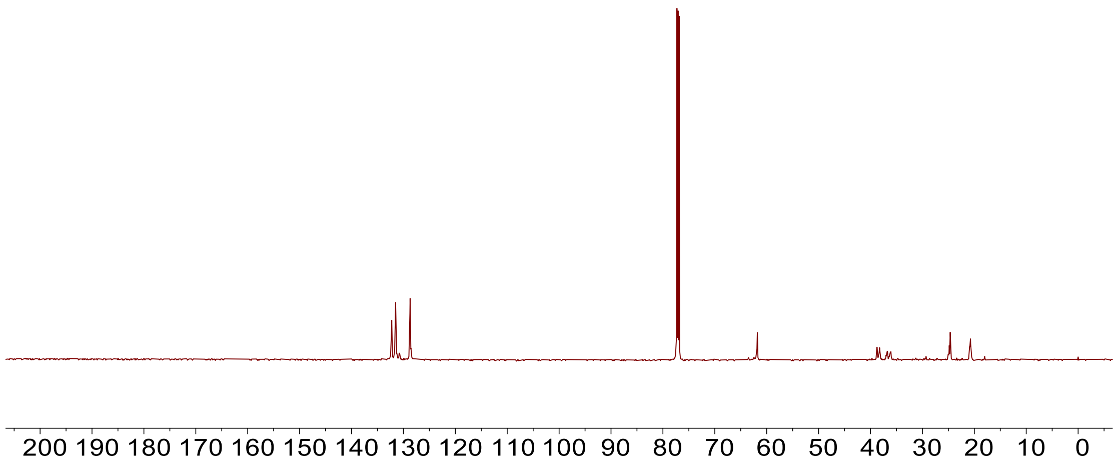
200 190 180 170 160 150 140 130 120 110 100 90 80 70 60 50 40 30 20 10 0

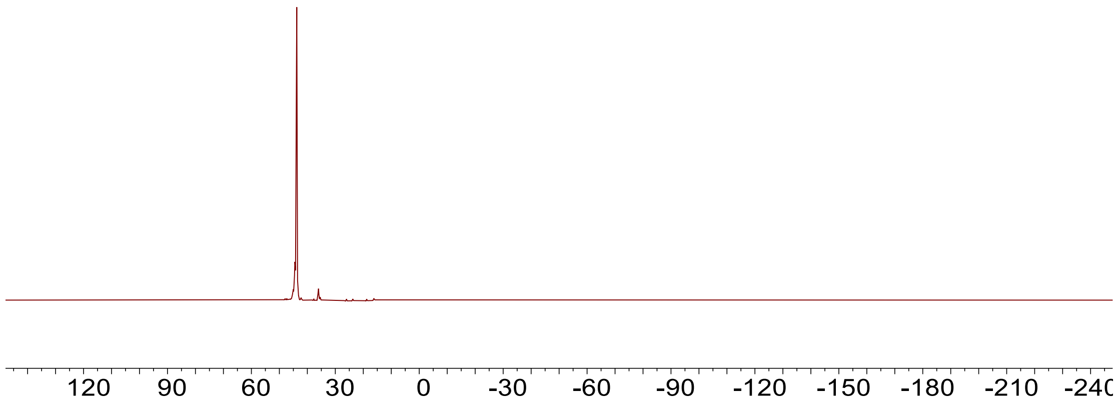
120 90 60 30 0 -30 -60 -90 -120 -150 -180 -210

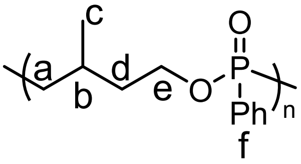

c
a
b
d
e
O
P
O
Ph
f
n

P4

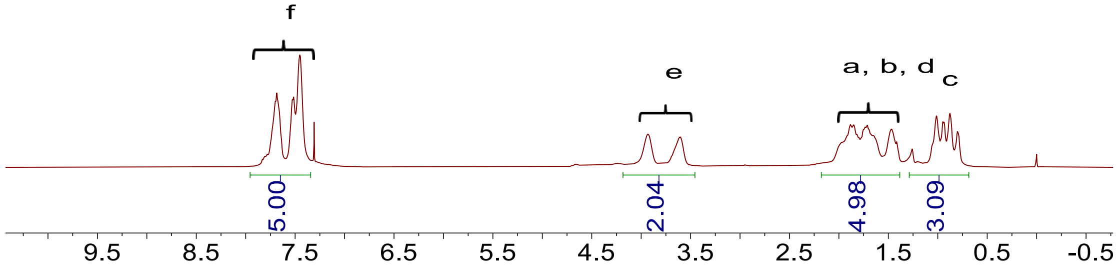

f
e
a, b, d
c
5.00
2.04
4.98
3.09
9.5
8.5
7.5
6.5
5.5
4.5
3.5
2.5
1.5
0.5
-0.5

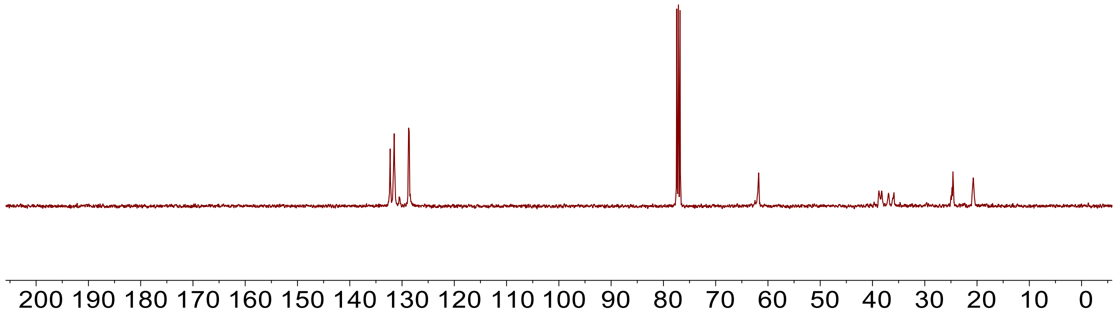

200 190 180 170 160 150 140 130 120 110 100 90 80 70 60 50 40 30 20 10 0

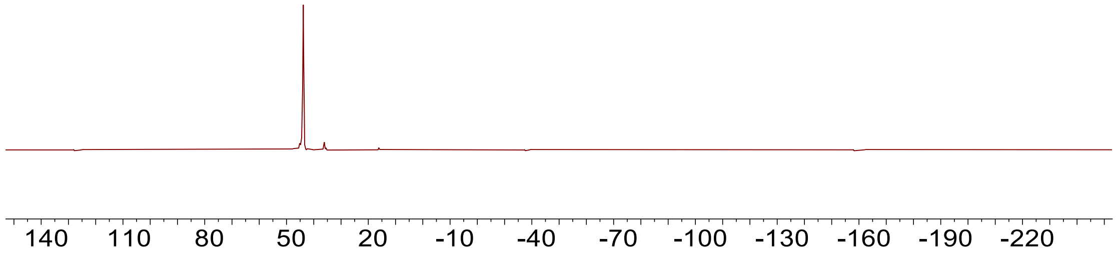
140 110 80 50 20 -10 -40 -70 -100 -130 -160 -190 -220

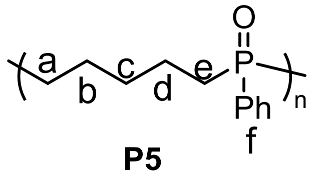
O
P
Ph
a
b
c
d
e
f
n

P5

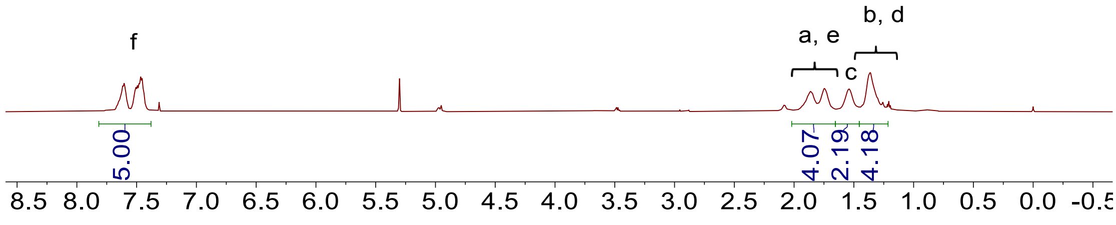
f
a, e
c
b, d
5.00
4.07
2.19
4.18
8.5 8.0 7.5 7.0 6.5 6.0 5.5 5.0 4.5 4.0 3.5 3.0 2.5 2.0 1.5 1.0 0.5 0.0 -0.5

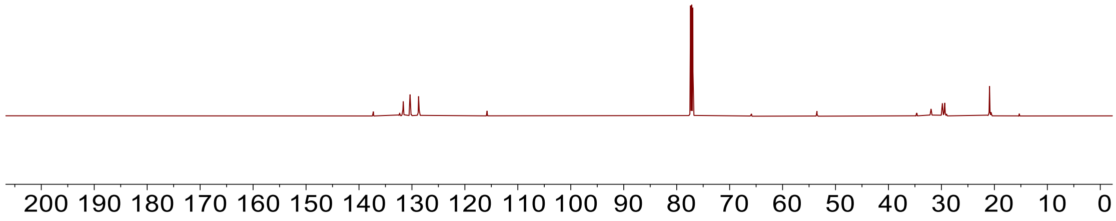
200 190 180 170 160 150 140 130 120 110 100 90 80 70 60 50 40 30 20 10 0

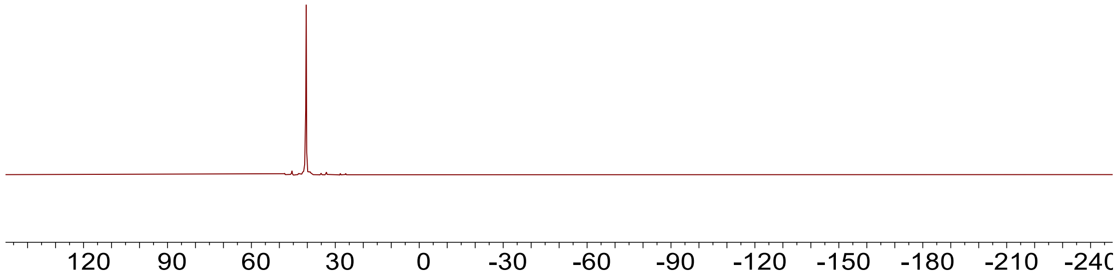
120 90 60 30 0 -30 -60 -90 -120 -150 -180 -210 -240

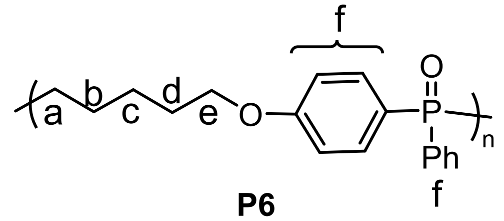
f
a
b
c
d
e
O
P
Ph
n
f

P6

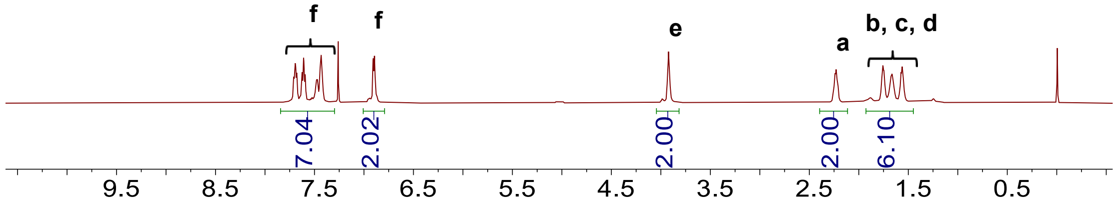
f
f
e
a
b, c, d
7.04
2.02
2.00
2.00
6.10
9.5 8.5 7.5 6.5 5.5 4.5 3.5 2.5 1.5 0.5

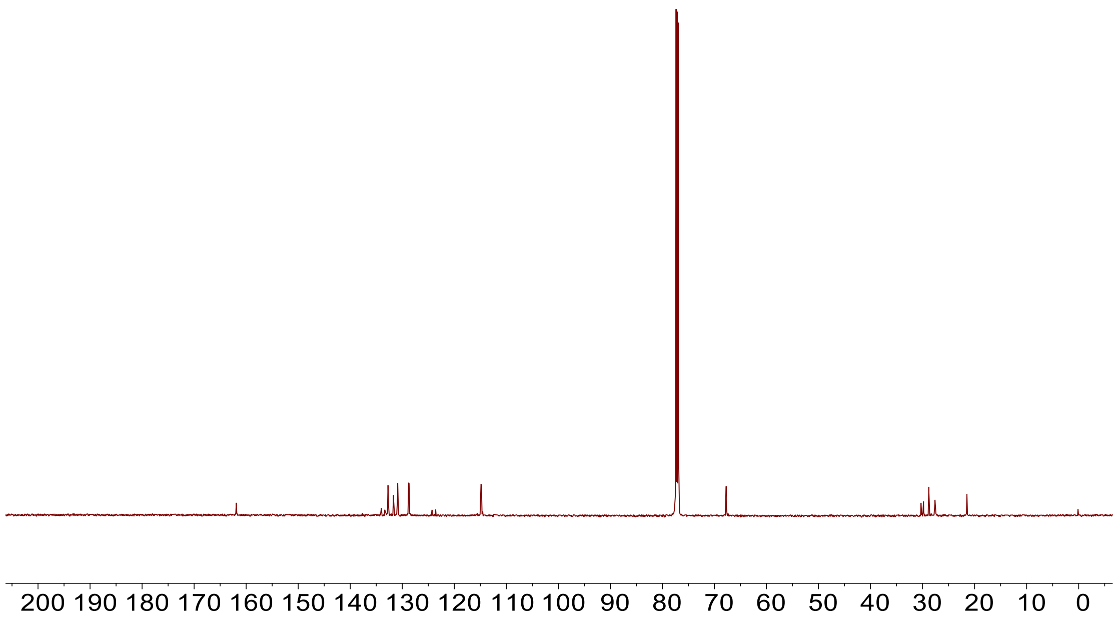
200 190 180 170 160 150 140 130 120 110 100 90 80 70 60 50 40 30 20 10 0

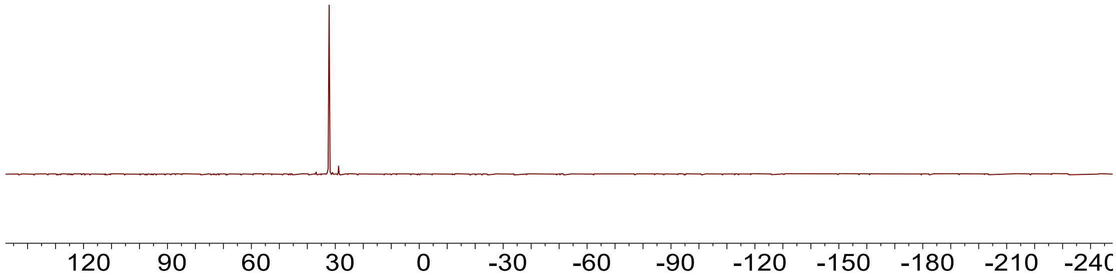
120
90
60
30
0
-30
-60
-90
-120
-150
-180
-210
-240

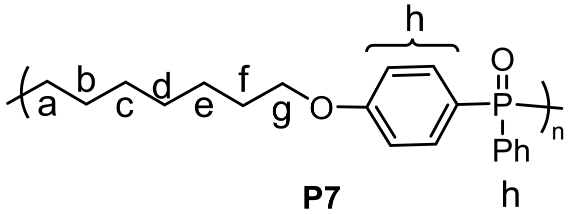
h
O
P
Ph
n
a
b
c
d
e
f
g
O
h

P7

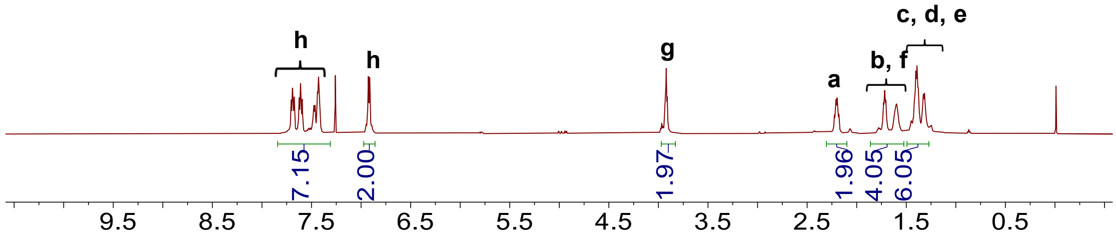
h
h
g
a
b, f
c, d, e
7.15
2.00
1.97
1.96
4.05
6.05
9.5
8.5
7.5
6.5
5.5
4.5
3.5
2.5
1.5
0.5

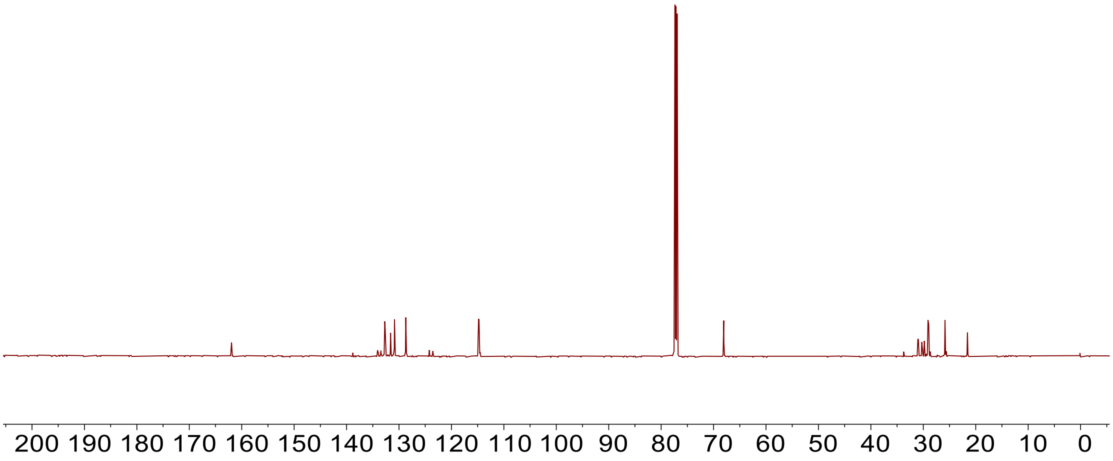
200 190 180 170 160 150 140 130 120 110 100 90 80 70 60 50 40 30 20 10 0

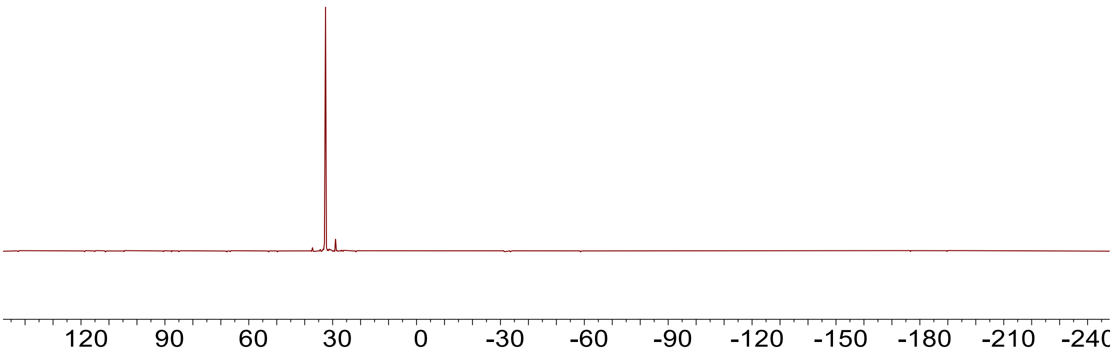
120 90 60 30 0 -30 -60 -90 -120 -150 -180 -210 -240

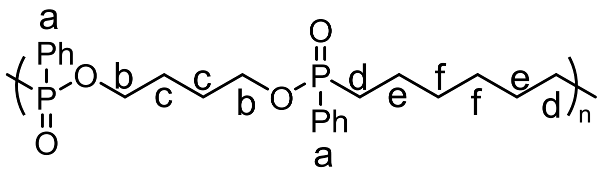
a
Ph
P
O
O
b
c
c
b
O
O
P
d
Ph
a
e
f
f
e
d
n

P8

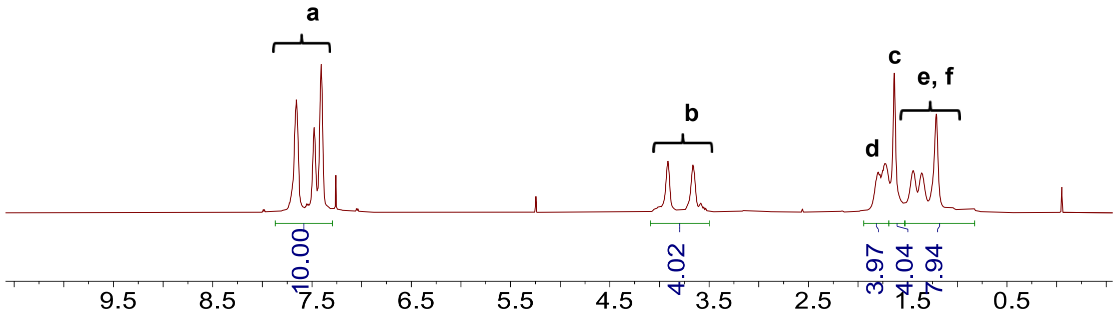
a
b
c
e, f
d
10.00
4.02
3.97
4.04
7.94
9.5
8.5
7.5
6.5
5.5
4.5
3.5
2.5
1.5
0.5

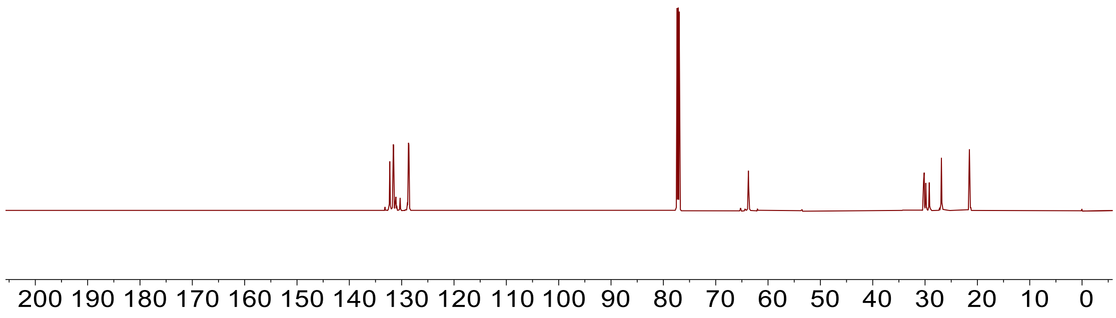
200 190 180 170 160 150 140 130 120 110 100 90 80 70 60 50 40 30 20 10 0

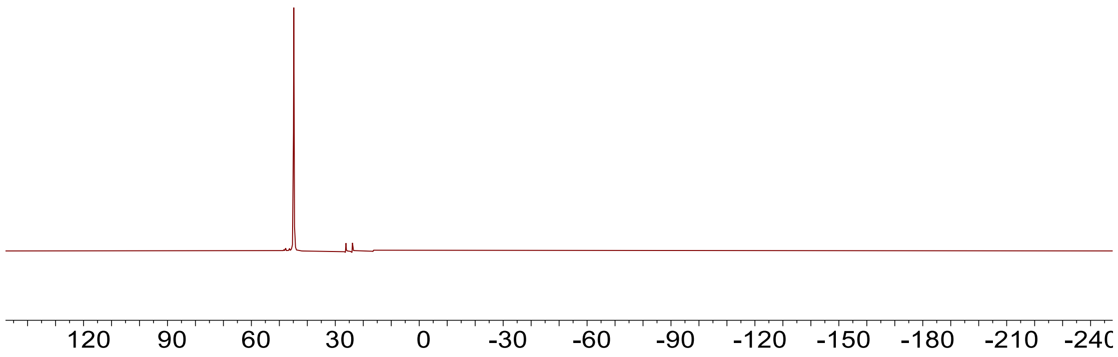
120
90
60
30
0
-30
-60
-90
-120
-150
-180
-210
-240

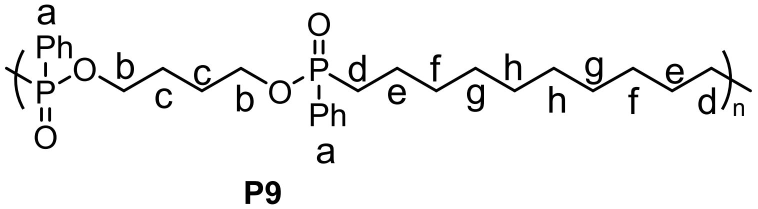
a
Ph
P
O
O
b
c
c
b
O
O
P
Ph
a
d
e
f
g
h
h
g
f
e
d
n

P9

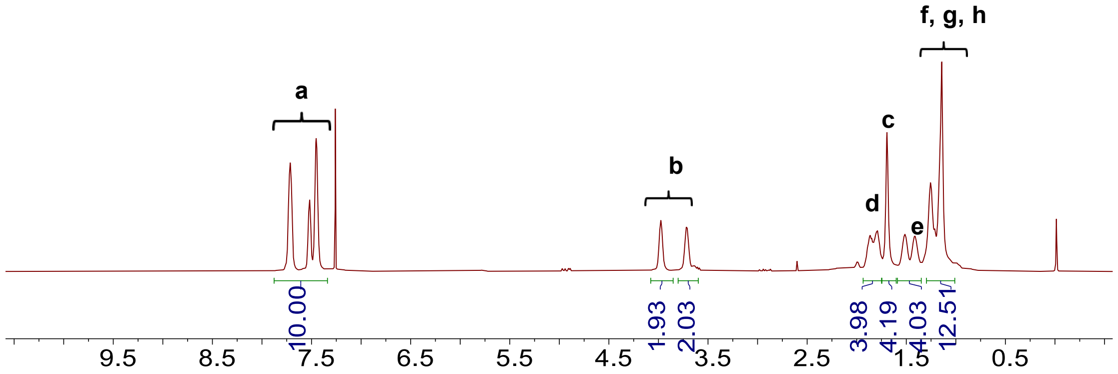
a
b
c
d
e
f, g, h
10.00
1.93
2.03
3.98
4.19
4.03
12.51
9.5
8.5
7.5
6.5
5.5
4.5
3.5
2.5
1.5
0.5

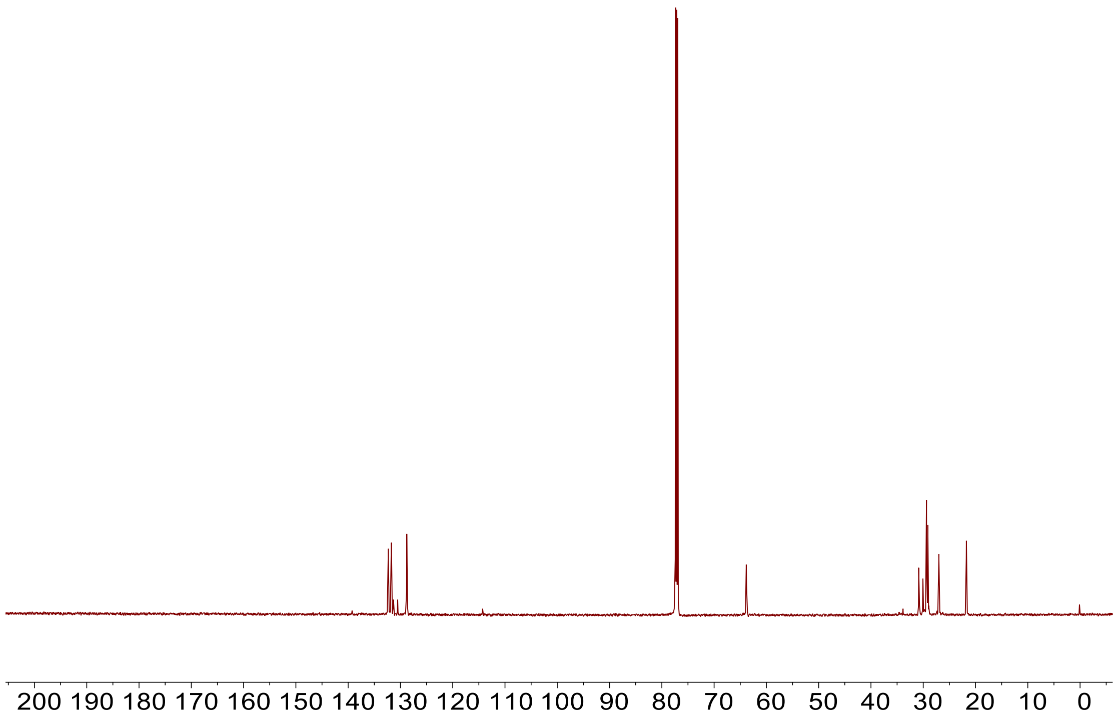
200 190 180 170 160 150 140 130 120 110 100 90 80 70 60 50 40 30 20 10 0

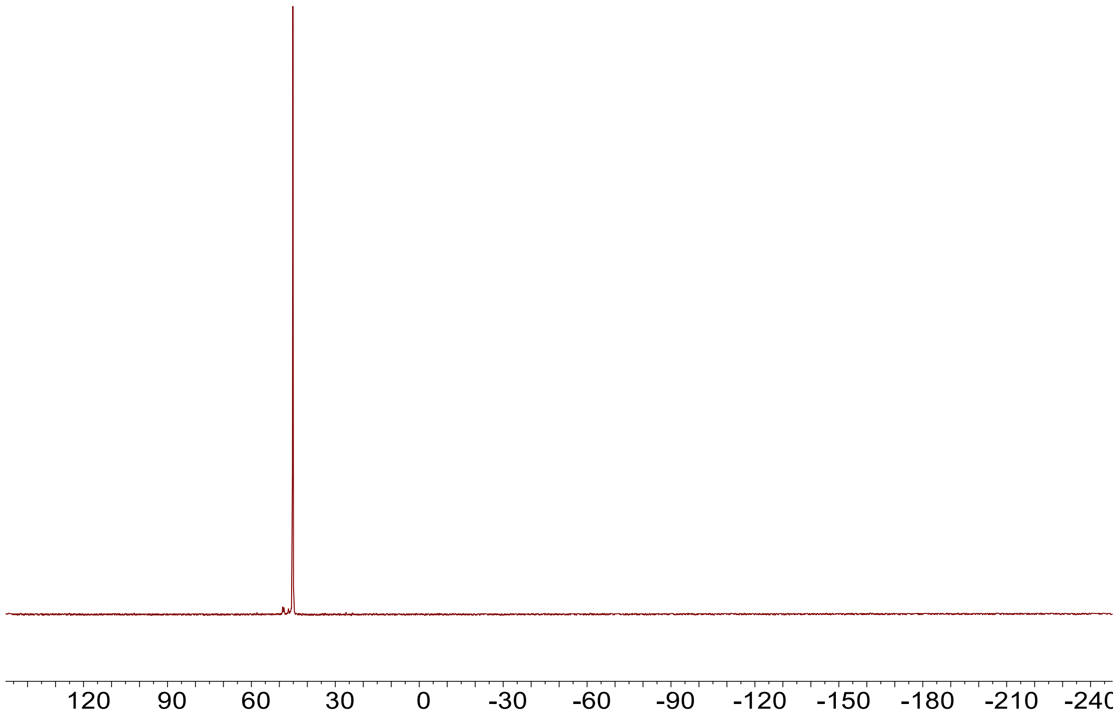
120 90 60 30 0 -30 -60 -90 -120 -150 -180 -210

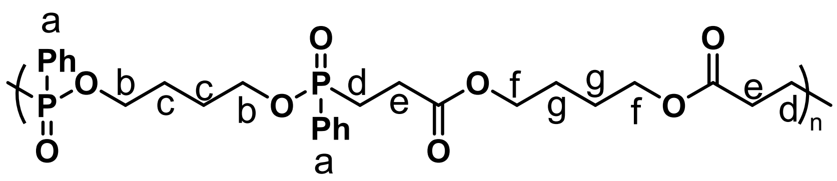
a
Ph
P
O
O
b
c
c
b
O
P
O
d
Ph
a
e
O
O
f
g
g
f
O
O
e
d
n

P10

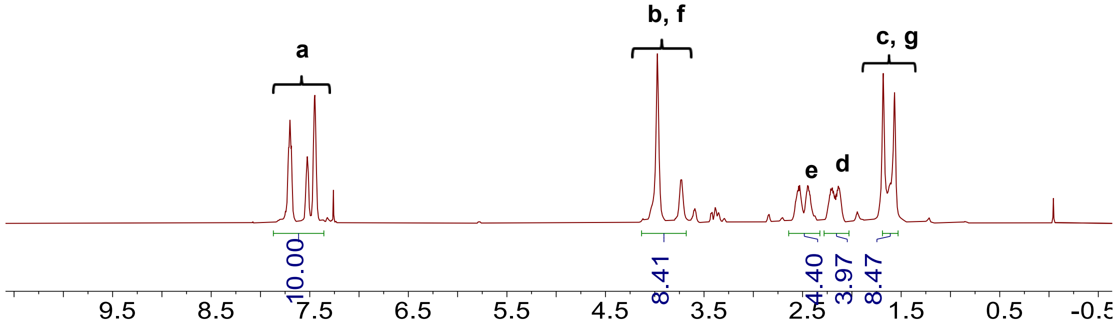
a
b, f
e
d
c, g
10.00
8.41
4.40
3.97
8.47
9.5
8.5
7.5
6.5
5.5
4.5
3.5
2.5
1.5
0.5

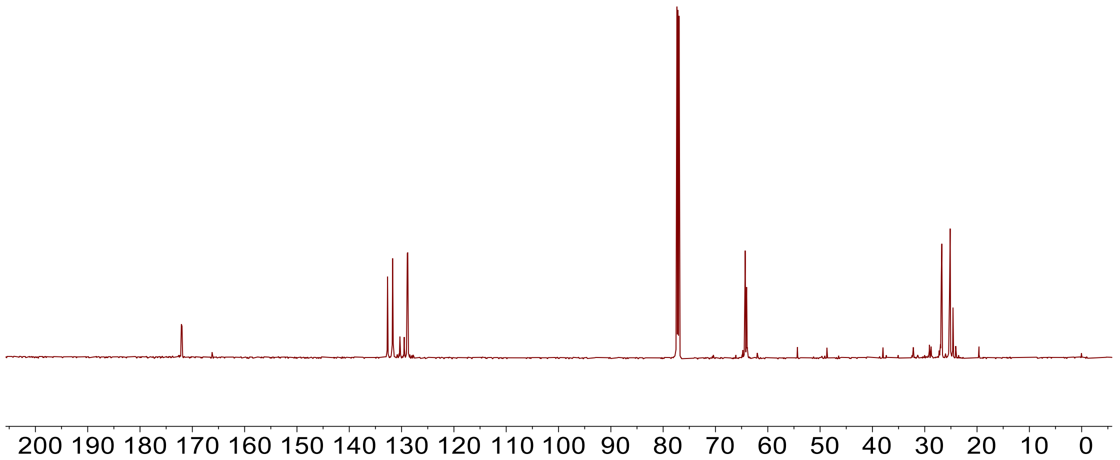
200 190 180 170 160 150 140 130 120 110 100 90 80 70 60 50 40 30 20 10 0

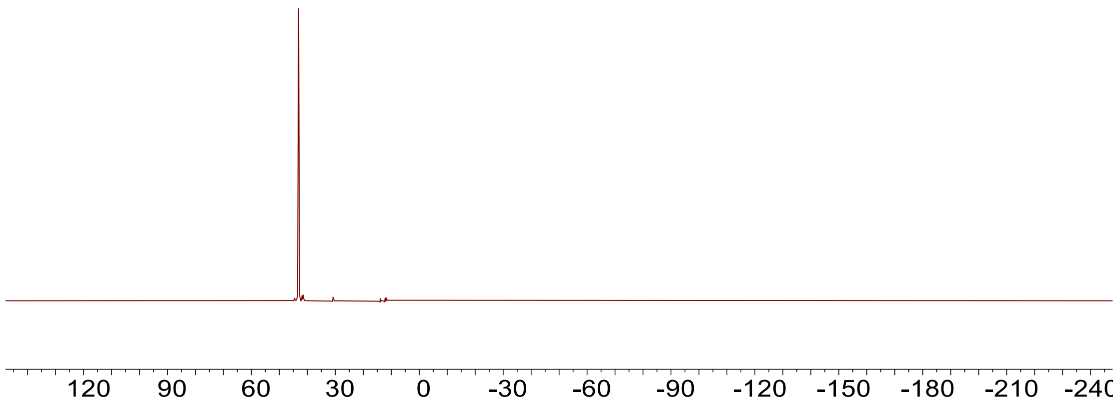

120
90
60
30
0
-30
-60
-90
-120
-150
-180
-210
-240

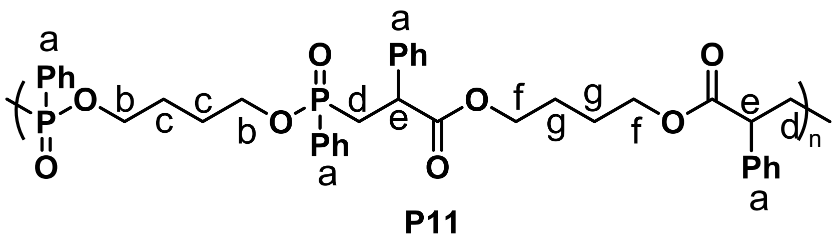

P11

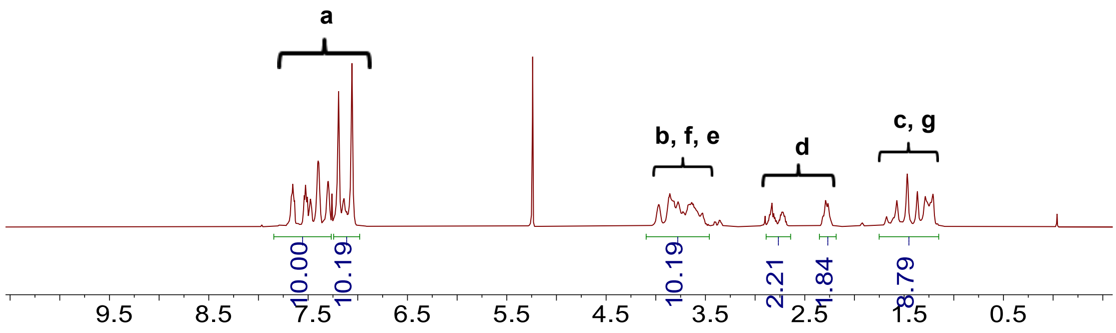

a
b, f, e
d
c, g
10.00
10.19
10.19
2.21
1.84
8.79
9.5
8.5
7.5
6.5
5.5
4.5
3.5
2.5
1.5
0.5

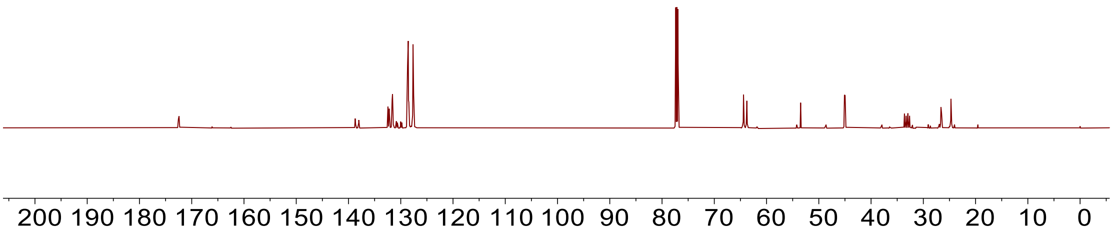

200 190 180 170 160 150 140 130 120 110 100 90 80 70 60 50 40 30 20 10 0

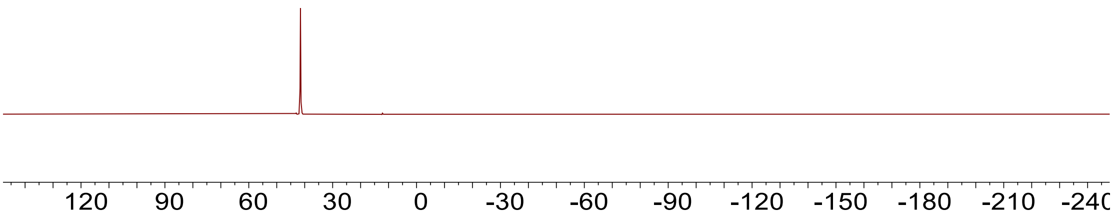

120 90 60 30 0 -30 -60 -90 -120 -150 -180 -210 -240

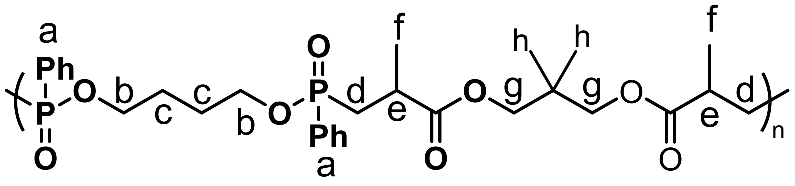
a
Ph
P
O
O
b
c
c
b
O
P
O
Ph
a
d
f
e
O
O
g
h
h
g
O
O
f
e
d
n

P12

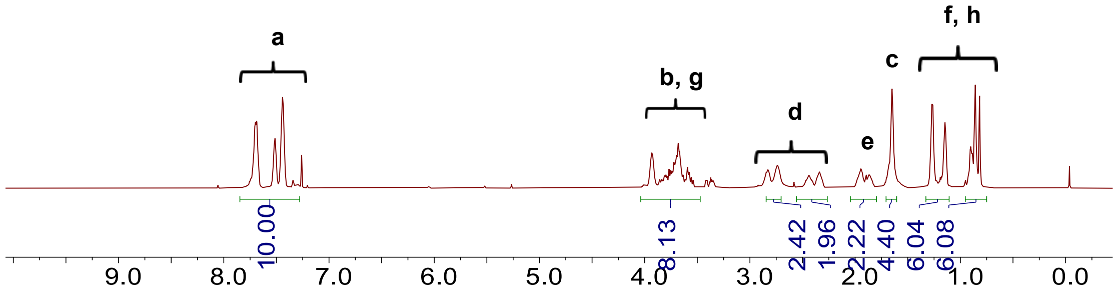
a
b, g
d
e
c
f, h
10.00
8.13
2.42
1.96
2.22
4.40
6.04
6.08
9.0
8.0
7.0
6.0
5.0
4.0
3.0
2.0
1.0
0.0

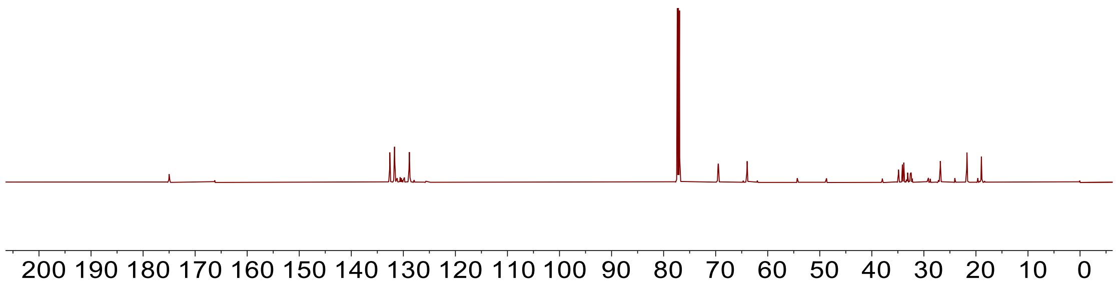
200 190 180 170 160 150 140 130 120 110 100 90 80 70 60 50 40 30 20 10 0

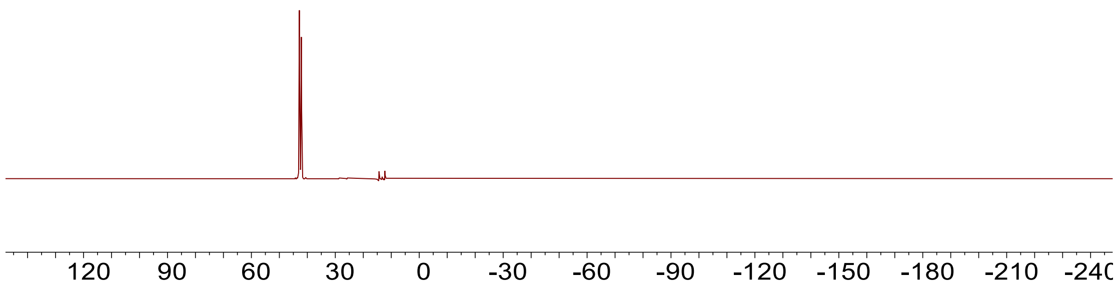
120
90
60
30
0
-30
-60
-90
-120
-150
-180
-210
-240
